# Assessing the Persistence and Mobility of Organic Substances to Protect Freshwater Resources

**DOI:** 10.1021/acsenvironau.2c00024

**Published:** 2022-08-02

**Authors:** Hans Peter H. Arp, Sarah E. Hale

**Affiliations:** † Norwegian Geotechnical Institute (NGI), P.O. Box 3930, Ullevål Stadion, NO-0806 Oslo, Norway; ‡ Department of Chemistry, Norwegian University of Science and Technology (NTNU), NO-7491 Trondheim, Norway

**Keywords:** persistence, mobility, environmental monitoring, drinking water, groundwater, hazard assessment, weight-of-evidence

## Abstract

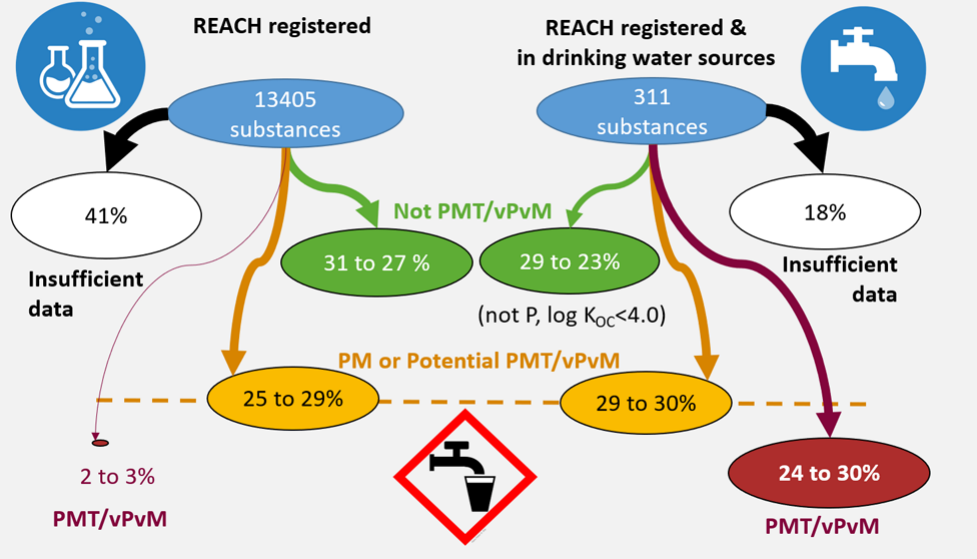

Persistent and mobile organic substances are those with the highest propensity to be widely distributed in groundwater and thereby, when emitted at low-levels, to contaminate drinking water extraction points and freshwater environments. To prevent such contamination, the European Commission is in the process of introducing new hazard classes for persistent, mobile, and toxic (PMT) and very persistent and very mobile (vPvM) substances within its key chemical regulations CLP and REACH. The assessment of persistence in these regulations will likely be based on simulated half-life, *t*_1/2_, thresholds; the assessment of mobility will likely be based on organic carbon–water distribution coefficient, *K*_OC_, thresholds. This study reviews the use of *t*_1/2_ and *K*_OC_ to describe persistence and mobility, considering the theory, history, suitability, data limitations, estimation methods, and alternative parameters. For this purpose, *t*_1/2_, *K*_OC_, and alternative parameters were compiled for substances registered under REACH, known transformation products, and substances detected in wastewater treatment plant effluent, surface water, bank filtrate, groundwater, raw water, and drinking water. Experimental *t*_1/2_ values were rare and only available for 2.2% of the 14 203 unique chemicals identified. *K*_OC_ data were only available for a fifth of the substances. Therefore, the usage of alternative screening parameters was investigated to predict *t*_1/2_ and *K*_OC_ values, to assist weight-of-evidence based PMT/vPvM hazard assessments. Even when considering screening parameters, for 41% of substances, PMT/vPvM assessments could not be made due to data gaps; for 23% of substances, PMT/vPvM assessments were ambiguous. Further effort is needed to close these substantial data gaps. However, when data is available, the use of *t*_1/2_ and *K*_OC_ is considered fit-for-purpose for defining PMT/vPvM thresholds. Using currently discussed threshold values, between 1.9 and 2.6% of REACH registered substances were identified as PMT/vPvM. Among the REACH registered substances detected in drinking water sources, 24–30% were PMT/vPvM substances.

## Introduction

The diversity of organic chemicals on the global market is continuously increasing,^1^ as are the number of substances being detected in freshwater resources.^2−5^ It is reasonable to hypothesize, based on these trends, that the diversity of substances appearing in freshwater resources will continue to increase, along with their total mixture concentration.^6,7^ This is a cause for concern for water quality. Once trace levels of contaminants become ubiquitous in a population’s water supply, population level effects may follow.^8,9^ If these same contaminants are persistent, effects can occur over long, intergenerational time scales.^10^ The current scale of exposure to contaminants in drinking water and other freshwater resources is only partly known. Many parts of the world do not have advanced water purification technologies to deal with diverse organic chemical pollutants,^11^ nor do they have active drinking water monitoring programs capable of identifying emerging substances. Recent high-resolution, nontarget approaches are helping to identify many previously unknown substances in freshwater resources, but at the same time these methods also indicate the presence of an even larger number of substances, such as transformation products, that are unknown and need to be identified.^4,5,12,13^ For these reasons, researchers and regulators are currently focusing on understanding the identity, sources, distribution, and uses of the diverse organic chemicals that are increasingly detected in drinking water sources, particularly those that are persistent and mobile.

Persistent and mobile organic substances^14^ are those that have the greatest propensity to contaminate water resources over large spatial scales when they are released in to the environment, even at low-levels. This is because, as the name implies, they are poorly degraded in the environment after emissions (persistent) and can be transported efficiently in aquatic systems and the subsurface (mobile). Of course, water resources can be polluted by substances that are not persistent and mobile, owing to factors such as close proximity of emissions to a water source or high volume, ubiquitous emissions (e.g., with substances like caffeine, benzene, etc.^15^ as will be explained more below). However, persistent and mobile substances may appear in water supplies even if emissions are relatively low and they occurred far away and a long time ago. An example of this is perfluorooctanesulfonate (PFOS) and other per- and polyfluoroalkyl substances (PFAS) that can spread from contaminated soil to aquifers and then to drinking water decades to centuries after restrictions are put in place.^14,16^

To address this, the European Commission has announced it will introduce persistent, mobile, and toxic (PMT) and very persistent, very mobile (vPvM) as new hazard classes for the CLP Regulation (Classification, Labeling and Packaging, Reg. (EC) 1907/2006) as well as the REACH Regulation (Registration, Evaluation, Authorisation and Restriction of Chemicals, Reg. (EC) 1272/2008).^17,18^ This would pave the way for the adoption of such classes into the United Nation’s Globally Harmonized System of Classification and Labeling of Chemicals (GHS) and adaptation in other regions.^19^ How persistence and mobility hazard thresholds will be defined in such regulations is currently being discussed, based on work in recent years by scientists and regulators.^20^

In this work, we present an overview of this recent discussion by exploring the threshold criteria for persistence and mobility based on chemical property data, including measured data, estimated data, and screening parameters. To do so, we first review the origins of the development of criteria for persistence and mobility. Then, these criteria are applied to substances registered under REACH, including known transformation products thereof, along with substances that have been detected in various freshwater media (waste water treatment plant effluent, surface water, bank filtrate, groundwater, raw water, and drinking water). The suitability of applying experimental and estimated screening parameters to assess persistence and mobility are discussed. This knowledge is collectively used to develop guidance for persistence and mobility substance assessment, provide a list of substances that could be considered as PMT/vPvM based on the collected data, and discuss potential environmental implications.

## Background

### Thresholds for Persistence and Mobility

Explicitly defining a persistent, mobile substance using quantitative thresholds has been the focus of much recent discussion.^20−22^ In real world systems, the transport and exposure of pollution is dependent on both intrinsic physicochemical properties of the contaminant and the extrinsic properties of how these are manifested in the surrounding environment. Chemicals that are readily biodegradable (i.e., not persistent) in laboratories on the scale of days may still be transported long distances in groundwater on the scale of years,^15^ due to variations in microbiological communities and environmental conditions present in the subsurface.^23,24^ Further, some insoluble (i.e., nonmobile) chemicals could potentially enter a drinking water supply during a flooding event^25^ or nearby industrial emissions,^26^ bypassing typical subsurface groundwater routes or bank filtration. In the context of real world natural variability, typical or simulated environmental conditions are needed for benchmarking thresholds for persistence and mobility.

#### Persistence

Persistence (*P*) as a chemical property refers to the chemical’s degradation rate in one or more environmental compartment(s).^27−31^*P* is typically assessed based on single compartment half-lives under specified conditions that are simulated in the laboratory.^29,31^ Guidelines have been developed to measure single compartment half-lives in water, soil, and sediment under defined conditions (darkness, temperature, microbial activity, etc.) such as the OECD methods 307, 308, and 309.^30−32^ However, there have been several concerns raised about how error prone these methods can be when deriving half-lives in certain situations.^33−35^ Even if these methods were not error prone, there are two overarching criticisms of the use of simulated half-lives to define persistence. First is the practical one, that the methods are expensive and time-consuming. Second is that simulated half-lives present a simplification of the natural variability of the real world. Some soils can be biodegradation hot spots, and others barren.^36^ Half-lives are dependent on temperature,^37^ depth,^37^ nutrient loads,^36^ pH,^36^ oxygen levels,^38^ bioavailability, and nonextractable residues.^39^ Even though simulated half-lives are not representative for all global environments, they are still very useful for ranking the relative persistence of one substance against the other under controlled conditions.^30^ They serve as a way of benchmarking the hazard of persistence, as they are intrinsic, laboratory-based substance parameters. Further, it should be emphasized that persistency within a single-compartment can itself be a major cause of concern, based on several examples of accumulating, persistent substances leading to local or planetary-boundary threats for a variety of fate and exposure pathways.^40^

For a local risk assessment relating to a specific emission and exposure scenario, however, a substance’s “overall persistence”, *P*_OV_, would be a better parameter to assess risk. *P*_OV_ considers the half-life in each compartment and the partitioning and exposure across compartments (like air, water and soil). *P*_OV_, however, does not lend itself to being a hazard category, as it is dependent on emission scenarios and local environmental conditions and is extremely data intensive, requiring several single-compartment half-lives as input or benchmarking approaches based on monitoring data.^28−30^ By contrast, simulated half-lives do not have to consider emission scenarios or local environmental conditions to rank relative persistency between substances.

For the purpose of protecting drinking water sources and freshwater environments, *P* in the aquatic subsurface is the most relevant media for a hazard assessment, for several reasons: (i) groundwater and bank filtrate are important, self-filtering water transport routes to drinking water sources; (ii) half-lives are longer in the subsurface than in surface media (like surface soils or surface water), making it a more conservative estimate of persistence;^10,14,41^ (iii) groundwater itself is broadly considered a pristine water supply that is inherently worthy of protecting for future generations from persistent substances.^42,43^

#### Mobility

Mobility (*M*) in the subsurface is considered as the potential of a substance to be transported long distances by porewater flow. In a local environment, mobility depends on the persistence of the substance within the soil, the sorption capacity of the substance to the surrounding soils and sediments, and the hydraulic conditions (e.g., flow rate, rainfall).^44^ Sorption capacity is generally quantified using equilibrium distribution coefficients, *K*_D_, which is the equilibrium concentration of a substance in soil, sediment, or sludge (solid) phase (*C*_solid_, μg/kg_solid_) to the that of the (pore)water phase (*C*_water_, μg/L_water_); see eq 1a. For organic substances, the *K*_D_ is often normalized to the mass fraction of organic carbon, *f*_OC_ (kg_OC_/kg_solid_), typically defined as all carbon that is not present as a carbonate, as presented in eq 1b.
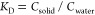
1a
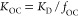
1b

Standardized methods to determine equilibrium *K*_OC_ (l/kg_OC_) at defined conditions have been developed. These methods include batch tests where a mixture of solids and water are spiked with a substance and mixed until equilibrium is reached (e.g., OECD 106),^45^ measuring the substance retention time in HPLC columns that have been correlated with a *K*_OC_ value for *neutral* organic substances (e.g., OECD 121),^46^ as well as several methods by the US-EPA (EPA OPPTS methods 835.1110, 835.1220, 796.2750).

The use of *K*_OC_ has been favored historically for comparing mobility data and conducting exposure assessments for neutral, organic substances,^47^ because the organic carbon phase is widely considered the dominating sorption component of soils, sediments, and sludges.^48,49^ This normalization allows laboratory determined *K*_OC_ values under defined conditions to be considered an intrinsic, laboratory-based substance parameter; however, because organic carbon itself is a heterogeneous environmental phase, some statistic distribution in *K*_OC_ values is to be expected considering diverse types of organic carbon. This statistic distribution can be particularly large in the case of charged and ionizable compounds, where *K*_OC_ is dependent not only on the organic carbon content but also on the concentration of contamination (nonlinear sorption) and on fluctuations in pH that affect the ionizability of soil and the analyte.^50^ In addition, the *K*_OC_ data can be biased by the ion-exchange interactions of minerals,^51^ competition effects with counterions,^51^ the presence of strong sorbents like black carbon and tars,^52^ weathering effects that create nonexchangeable residues,^53^ sorption hysteresis,^54^ enrichment of surfactants at the air–porewater interface,^55,56^ coagulation with humic matter,^57^ sorption site and pore-blocking by organic matter,^58^ in addition to the heterogeneity in types of organic carbon present.^59^ All these complex effects are extremely important for risk assessments carried out at a local scale yet are also challenging to fully account for due to their complexity. However, for a generic ranking or benchmarking of the hazard of mobility in all (globally occurring) soil types, it is sufficient to measure *K*_OC_ for various soil/sediment types, using a standardized test procedure (e.g., OECD 106 or equivalent) over a range of porewater conditions (e.g., pH) and then make a comparison of the statistical distribution of these values.^60,61^

#### Persistent and Mobile

**Figure 1:**
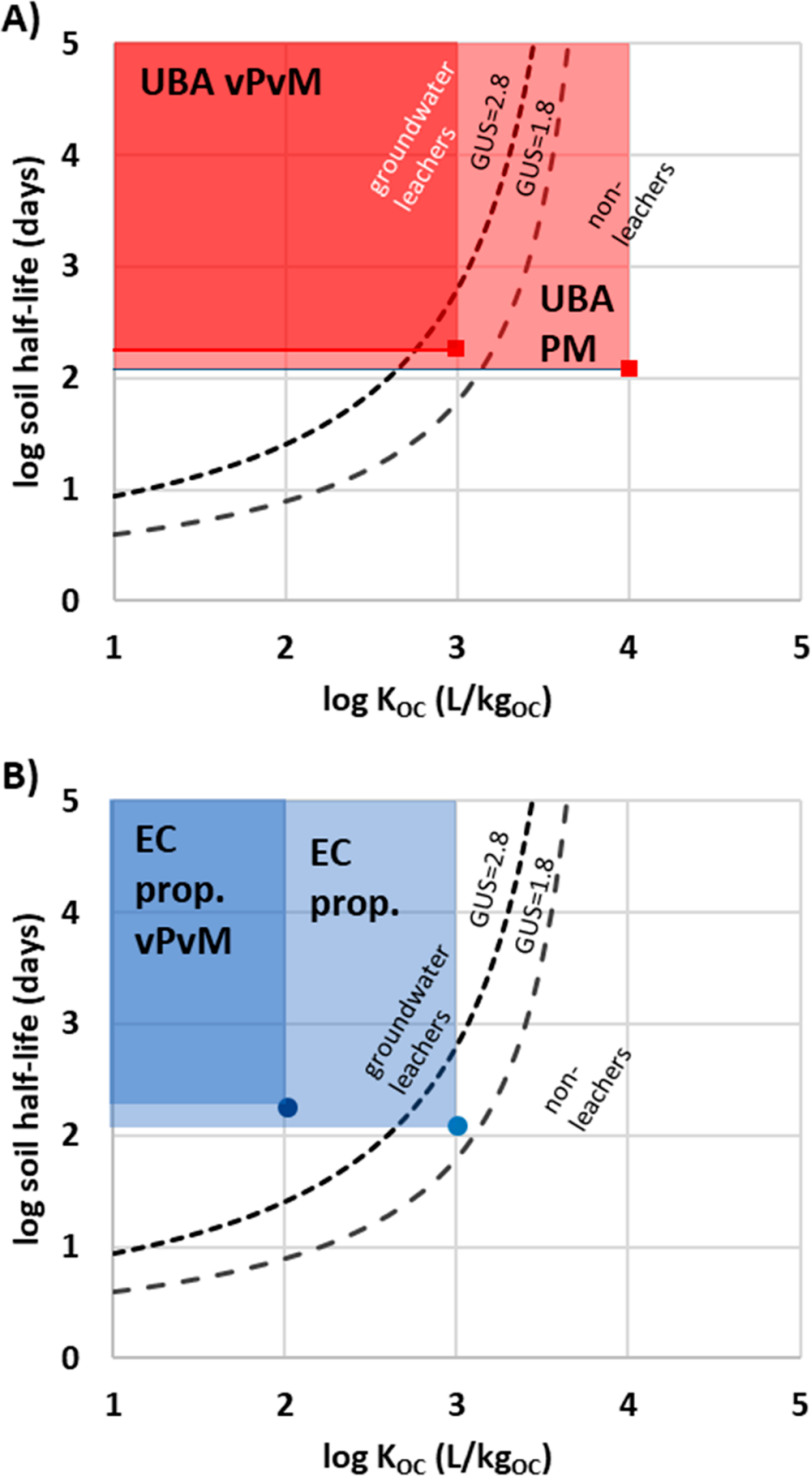
GUS plots of soil half-life vs log *K*_OC_ showing different criteria for persistent, mobile substances, including the GUS index of <1.8 for nonleachers in groundwater and >2.8 for leachers in groundwater. (A) Mobility criteria developed by the German Environment Agency (UBA) for soil and sediment (PM: half-life > 120 days, log *K*_OC_ < 4.0; vPvM: soil half-life > 180 days, log *K*_OC_ < 3.0). (B) Mobility criteria currently proposed by the European Commission (EC) for inclusion in the CLP regulation (PM: half-life > 120 days, log *K*_OC_ < 3.0; PM: soil half-life > 180 days, log *K*_OC_ < 2.0).

The “Groundwater Ubiquity Score” or GUS, developed by Gustafson in 1989,^62^ was an early and influential approach to identify persistent and mobile substances based on soil-half-lives, *t*_1/2,soil_, and *K*_OC_ (eq 2).
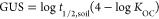
2When applying this equation, substances with GUS < 1.8 were considered as being a “nonleacher” to groundwater, and those with a GUS > 2.8 were considered a “leacher” that can contaminate groundwater (Figure 1). This type of conceptualization of persistency and mobility has been used in various forms. In Europe, an important application is the guidance on the Biocidal Products Regulation (EU 528/2012) that uses thresholds of *t*_1/2,soil_ > 21 days and *K*_OC_ < 500 L/kg_OC_, which would correspond to a GUS of 1.7 (or just across the border of being a “nonleacher”), for whether groundwater impacts need to be assessed.^63^ Similarly, the United Nation’s Food and Agricultural Organization uses *t*_1/2,soil_ and log *K*_OC_ values to characterize the degree of degradability and mobility in soil.^64^

More recently, the German Environment Agency (in German: *Umweltbundesamt*, UBA) in 2019 introduced the use of a combination of half-lives and log *K*_OC_ as part of the criteria to identify PMT/vPvM substances under REACH.^20^ A key difference regarding the definition of persistency used in the GUS and the proposed PMT/vPvM criteria is that the later broadens the definition of persistency from just soil to other media (i.e., fresh and marine water and sediments) to be more consistent with the definition of persistent (P) and very persistent (vP) used under the European REACH regulation^31^ as well as the Stockholm Convention criterion for Persistent Organic Pollutants (POPs).^31,65^ A similar approach for including this extended definition of P and vP was also used by the European “Voluntary Groundwater Watch List Concept & Methodology”^66,67^ and in the European Commission (EC) proposal in 2021 for the PMT/vPvM criteria in the CLP Regulation.^17,18^ The practical justification for this approach is that it allows regulatory definitions and guidelines developed for persistency assessments in different environmental media to be harmonized and directly transferable to the PMT/vPvM hazard assessment criteria. The theoretical justification for taking this approach is that substances that are persistent in soil are often persistent in other media as well,^68^ though with some exceptions such as when soil persistency tests are influenced by nonextractable residues.^39^ The PMT/vPvM criteria proposed in 2019 by UBA and in 2021 by the EC also differ from GUS in that they present fixed P/vP and *K*_OC_ values as thresholds, unlike GUS which uses these parameters as variables in a threshold-function (eq 2, Figure 1). The thresholds of the proposed UBA criteria from 2019 are minimum, experimentally measured log *K*_OC_ values determined at a pH between 4 and 9 of <4.0 as Mobile (M) and of <3.0 as very Mobile (vM). At the time of writing (April 2022), the proposed EC log *K*_OC_ thresholds are less stringent for M and vM and are <3.0 and <2.0, respectively. Both proposals use the same P and vP cutoffs for soil of *t*_1/2,soil_ >120 days and >180 days, respectively. These cutoffs are compared on a GUS score chart in Figure 1.^18^

As is evident from Figure 1, substances meeting the proposed EC PMT and vPvM substance thresholds and UBA vPvM substance threshold would be considered “groundwater leachers” according to the GUS score. Substances meeting the UBA PMT substance threshold would include “nonleachers” according to the GUS score. The justification for considering GUS score “nonleachers” as UBA PMT substances was to account for the many persistent and toxic substances that have been detected in groundwater and drinking water, or able to penetrate bank-filtration systems, with a log *K*_OC_ between 3.0 and 4.0.^21^

### Screening Parameters for Persistence and Mobility

Using the threshold definitions presented above, classifying substances as persistent and mobile based on simulated half-lives and batch-test *K*_OC_ values has serious limitations in terms of data availability. Experimentally determined simulated half-lives are quite rare. In 2013, UNEP reported that only 220 out of 95 000 chemicals used by industry have experimentally determined biodegradation half-lives.^69^ To help compensate for this, the European Chemicals Agency (ECHA) developed guidelines to assess persistency based on screening tests and weight-of-evidence approaches for use when half-lives were lacking, such as the ability to conclude “not persistent” based on readily or inherently biodegradable screening tests.^32^ However, such screening tests cannot be used directly to conclude P or vP, but rather “Not Persistent” (Not P) or “Potential P/vP”. There are different types of data that can also be used in weight-of-evidence approaches, such as read-across methods and quantitative structure–activity relationships (QSARs) to predict half-lives, in addition to field measurements and observations.^32^

Experimental log *K*_OC_ data is also not available for all substances, particularly for ionic substances which can exhibit more variability across soils,^61^ as described above. More commonly available parameters that may correlate with log *K*_OC_ values, particularly for neutral, nonpolar substances, are the octanol–water partition coefficient for nonionizing organic compounds (*K*_OW_) and HPLC retention times (e.g., OECD 121). However, these parameters do not account for ionic interactions between organic compounds and soil, which can substantially alter the mobility of ionic species, as well as be influenced by pH, counterions in the porewater, and the heterogeneity of the soil and minerals present,^50,51,60,70−72^ as mentioned above. To partly address this, the octanol–water distribution coefficient for ionizable substances (*D*_OW_) can be used.^41^ However, this parameter just considers the solubility of the charged and neutral species at a specific pH, and not the pH dependence of the ionic interactions with the soil, so it is not appropriate as a proxy for log *K*_OC_.^61^ Nevertheless, it may still play a role as a screening parameter for prioritizing what charged or ionizable substances are potentially mobile.^61^

Herein the performance of using various screening parameters for half-lives (e.g., readily biodegradable tests, QSARs) and *K*_OC_ (i.e., using *K*_OW_ and *D*_OW_ values) is investigated empirically to assess their performance as screening parameters to identify PMT/vPvM substances.

## Materials and Methods

### REACH Database and Transformation Products

The list of REACH registered substances (https://www.echa.europa.eu/information-on-chemicals/registered-substances) was downloaded on September 19, 2019. At this time, it contained a total of 22 400 substance listings. After consulting various databases, as described below, at least one organic chemical constituent was identified in 15474 of these registered substances (with a known or provided structure). After checking the structural information (as described below), there was a total of 12 960 unique organic structures, 998 of which occurred in multiple REACH substances. The most commonly reoccurring substances with at least one carbon atom were acetate (in 61 substances), carbonate (in 54 substances), and toluene sulfonic acid (in 38 substances).

To identify transformation substances of REACH registered compounds, lists of experimentally demonstrated transformation pathways were utilized from the EAWAG-BBD database (http://eawag-bbd.ethz.ch/, January 26, 2016 version), the EAWAG-soil database,^73,74^ and the SwissPest19 database.^4,75,76^ These databases mainly included pharmaceutical substances; nevertheless, there were matches with 1066 REACH registered substances, that were collectively found to be parents of 617 unique transformation products. Of these, 172 were already found in the REACH registered database. The most common transformation products were oxidized benzene rings (catechol, hydroquinone, hydroxybenzoic acid) or small aliphatic chains (formaldehyde, acetaldehyde, etc.). The list of the 12 960 unique REACH registered substances and 445 unique transformation products can be found in the Supporting Information as part of the large data set in Table S1.

### Chemical Structure Identification

Chemical structures for all substances were obtained by compiling Simplified Molecular Input Line Entry System (SMILES) codes from the following sources, in order of priority. First, available and quality-controlled SMILES for REACH registered substances EC-numbers from an earlier study was used.^41^ For the remaining substances, chemical structure information was obtained from the QSAR Toolbox structure database (https://qsar-toolbox.org/, accessed October 1, 2020) and an IUCLID database (i.e., what REACH registrants provided, https://iuclid6.echa.europa.eu/de/reach-study-results, downloaded prior to this study in April 2017), and if information was still missing, the ChemAxon “Name to Structure” converter (https://www.chemaxon.com/, accessed September 22, 2019) was used to convert CAS numbers and common names to structural information. Structures from QSAR toolbox, IUCLID, and ChemAxon’s “Name to Structure” were then processed using the Open Babel software^77^ (available from http://openbabel.org/wiki/Main_Page) to convert all structural information into SMILES codes with the same dative format as well to International Chemical Identifier codes (InChI) and InChIKey codes. REACH substances that contained no carbon atoms (1002 substances) or those for which no structure information was provided/available (6668 substances) were excluded. To automatically identify inconsistently reported structures or incorrect structures across databases, a topographical analysis was used to flag the following: (1) differences in number of elements (i.e., the number of carbons, oxygens, etc. should match across the different SMILES database for a given CAS number) and (2) differences in net the charge of the structure (net charge of all positive and minus charges should be zero). In cases of mismatches between elements or net charge, the structures were manually checked to see if one of the provided/predicted structures was clearly wrong (i.e., text entries instead of SMILES codes). In cases where this was not clear, manual comparisons were done with the web site PubChem to choose the best structure. Structures were classified as pseudo-organic (just one carbon atom), organic (more than one carbon atom), organoborane (organic with at least one boron), organosilicon (organic with at least one silicon), or organometallic (organic structure with one other atom other than H, B, N, O, S, P, Si, or a halogen). Collectively, these are referred to as “organic structures”, and they were included in the PMT/vPvM substance assessment. Other molecules with no structure, inorganic, or carbonaceous solids (e.g., activated carbon, charcoals), and carbides were excluded from further consideration.

As the REACH database consisted of several complex substance mixtures, a system of structural quality flags was utilized to indicate that the obtained chemical structure may be of low quality. A structure could have one or more of these structural flags, which were as follows: ***charge balance***, in cases where the positive and negative charges on the structure did not cancel out due to, e.g., counterions not being provided (285 structures); ***reaction product***, in cases when the parent substances to a reaction was reported, but not the actual reaction products (83 unique structures across 329 substance entries); ***petro***, in cases where the substances were distillates of petroleum products according to their name (48 unique, proxy structures identified across 212 substance entries); ***residues***, in cases where the word “residue” was in the name, excluding petroleum distillates (22 substances entries); ***mixture***, for substances that were loosely defined mixtures, in which the name contained words like “derivatives”, “branched”, “isomers”, “ethoxylated”, “and”, “of”, or plural forms of chemical names (e.g., ethers, alcohols) (207 unique proxy structures across 2522 substance entries); ***extracts***, in cases where a substance contained the word “extract” in its name (5 unique structures). In cases of defined mixtures, where it was explicitly stated what chemicals were present, such as cations and anions in salts, or mixtures of defined chemicals, one EC number could be associated with more than one unique organic chemical structure. Tautomerism and stereoisomerism was not explicitly checked for. The database of all unique structures identified by this methodology, along with structural quality flags, are presented in the Supporting Information (Table S1).

### Detected Substances in Freshwater

Monitoring studies of organic chemicals in the following aquatic media were collected from the literature: wastewater treatment plant effluent (WW), surface water (SW), bank filtrate (BF), groundwater (GW), raw water (RW), and drinking water (DW). This was done by using the search terms “organic chemical”, “contaminant”, and the name of the media, with the years 2000–2019, using Google Scholar (scholar.google.com, last accessed December 2019) and Clarivate Web of Science (webofscience.com, last accessed December 2019). As the focus was on detected substances, no search filter was applied for geographical region, water treatment technology, or local hydrogeological conditions. The aim of the literature search was not to be comprehensive and compile every substance ever detected in freshwater, but rather we sought to assemble a sufficiently large database of detected substances to probe the distribution of their persistence and mobility properties. For this reason, monitoring studies with large numbers of organic chemicals and compilations of such studies were primarily consulted. In total, 55 unique monitoring studies or compilations thereof were included, many of which contained data for multiple aquatic media of interest. There were 12 sources for WW,^78−89^ 6 for SW,^2,80,90−93^ 7 for BF,^80,91,93−97^ 15 for GW,^2,5,80,91,98−108^ 6 for RW,^15,109−113^ and 22 for DW.^42,80,91−93,101,102,107,114−125^

As presented in Table 1, 1289 unique organic chemicals were detected across these 6 types of water media. The greatest number of unique organic chemicals detected were for SW, totalling 1021, due to the availability of comprehensive compilation studies.^80,90^ In comparison, the fewest unique structures were detected in bank filtrate (*n* = 114) and the second fewest in raw water (*n* = 212), which coincided with comparatively fewer studies being available for these media. For groundwater and drinking water, 338 and 385 unique substances were found to be detected, respectively, based on the literature review.

**Table 1:** Overview of the Number of Monitoring Studies Considered in This Study and Unique Chemicals Detected in Different Freshwater Media

media	no. of sources	unique organic chemicals detected	of which are REACH registered	of which have REACH volumes in 2019 of >10 tons/annum
WTP effluent	12	442	143 (32%)	30 (7%)
surface water	6	1021	387 (38%)	172 (17%)
bank filtrate	7	114	60 (53%)	25 (22%)
groundwater	15	338	165 (49%)	80 (24%)
raw water	6	212	125 (59%)	64 (30%)
drinking water	22	385	186 (48%)	90 (23%)
all considered media	55	1289	509 (39%)	229 (18%)

Considering all water media, 509 of the 1289 substances detected in water were REACH registered substances. The entire number of unique chemical structures considered in this study is 14 203, with 12 960 being REACH substances, 445 being unique transformation products, and 798 being the monitored substances which were not REACH registered.

### PMT/vPvM Hazard Assessment

**Figure 2:**
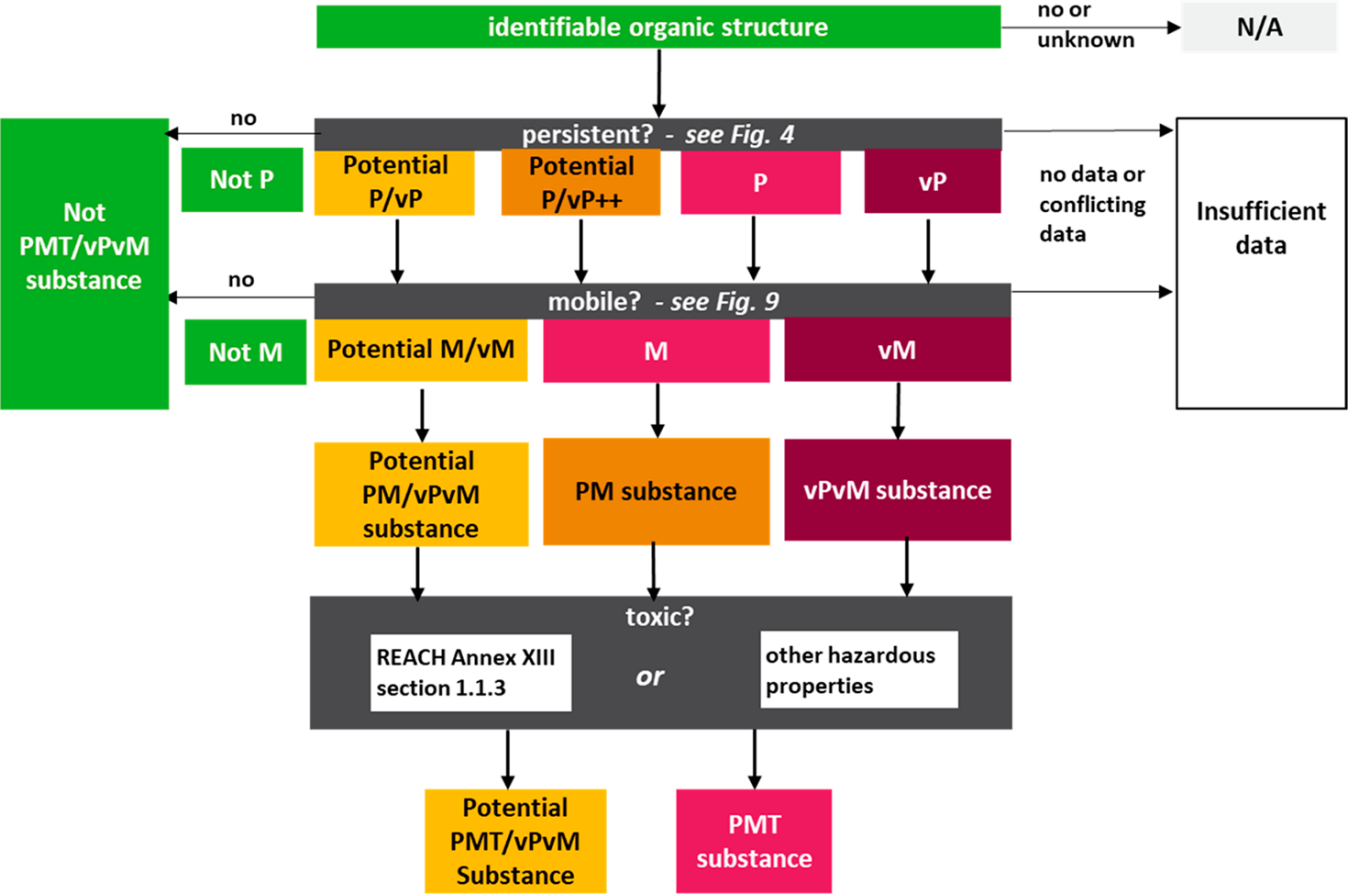
Overview of the assessment procedure to identify PMT/vPvM substances. First, if the substance contains an identifiable organic structure, it is assessed for persistence, with possible conclusions being very persistent (vP), persistent (P), Potential P/vP++ (very likely to meet the P or vP criteria), Potential P/vP (not readily/inherently biodegradable, but unknown if P/vP), and Not P. Unless the substance is “Not P” or there is "insufficient data" because of “no data or conflicting data” for persistency, it is assessed for mobility (with conclusions being very mobile (vM), mobile (M), potentially M/vM, and “not M”). Unless the substance is “not M” or there is "insufficient data" because of “no or conflicting data” for mobility, it is assessed for toxicity. Final conclusions can be “vPvM & PMT”, vPvM, PMT, “Potential PMT/vPvM”, PM, “Not PMT/vPvM”, or “insufficient data”. More information on the persistence and mobility assessment can be found in Figures 4 and 9.

The general overview for conducting a PMT/vPvM hazard assessment applied here, presented in Figure 2, is based on the workflow developed by the UBA,^20^ but is expanded to account for weight-of-evidence. Definitions of the PMT/vPvM hazard assessment conclusions are presented in Table 2.

**Table 2:** Criteria for Different Classifications Related to Persistence, Mobility, and Toxicity Used in This Study

category	criteria
vPvM	both *vP* and *vM* are met; alternatively, *Potential P/vP++* and *vM* with additional weight-of-evidence of *vP*
PM	either the combination of *P* and *M*, *vP* and *M*, or *P* and *vM* is met; alternatively, *Potential P/vP++* and *M/vM* with additional weight-of-evidence for the combination of *P* and *M*, *vP* and *M* or *P* and *vM*
PMT	A *PM* substance also meets the *T* criterion; if a *vPvM* substance meets the *T* criterion, it is considered *vPvM* & *PMT*
potential PM/vPvM	any combination of *Potential P/vP* with *M* or *vM*; *Potential P/vP++* with *M* or *vM* but no additional weight-of-evidence justifying PM, vPvM, OR any combination of P, vP, Potential P/vP, or Potential P/vP++ with Potential M/vM
not PMT/vPvM	any substance that is not P/vP or not M/vM; subcategories include “(Potential)P & not M”, meaning any substance that is vP, P, "Potential P/vP++", or "Potential P/vP" but is "Not M", and “Not P & Not M”
no or conflicting data	no data is available, only QSAR data are available but gives unclear predictions, or the structure provided for the substance is considered uncertain or inappropriate

Following the assessment procedure work flow in Figure 2, the substance itself is first evaluated to see if it contains an identifiable organic constituent (including mixture components, impurities, additives, and transformation products), as described above. For REACH registered substances with exclusively inorganic constituents or for those for which no organic structures were reported by the registrant, the substance was considered “not applicable” for further analysis, due to actual nonapplicability or lack of information, respectively. Following this, a P/vP assessment was conducted for all organic constituents, as described below. If a chemical constituent was assessed as P, vP, Potential P/vP++ (meaning very likely to meet either the P or vP criteria based on weight-of-evidence), or Potential P/vP (meaning not readily or inherently biodegradable, but unknown if it fulfils the P/vP criteria), it was then assessed for mobility. If it was assessed as “not P”, it was considered “not PMT/vPvM”, or if no data was available, the assessment was concluded as “no or conflicting data”. If a P, vP, Potential P/vP++, or Potential P/vP substance was considered “not M”, it was considered “not PMT/vPvM”. Alternatively, if constituents were assessed as “Potential M/vM” (meaning the data is not clear if the “Not M”, M, or vM criteria is met), it is considered either a “Potential PM/vPvM” substance or a “Potential PMT/vPvM” substance if toxic or potentially toxic. Otherwise, if a P, vP, or Potential P/vP++ substance meets the M or vM criteria in addition, it can be either a PM substance or a vPvM substance (subject to weight-of-evidence in the case of the Potential P/vP++ conclusion; see Table 2 for a further explanation). Finally, if a PM substance is considered toxic according to the REACH criteria or additional criteria,^20^ it is considered a PMT substance; if a vPvM substance is considered toxic, it is considered a vPvM and PMT substance.

#### Persistence Data and Evaluation

The data sources and procedures used to conduct the P/vP^21,32,41,91^ assessment herein were as follows, in order of priority: (1) Established P or vP classifications under Article 57 of REACH or by the Stockholm Convention. (2) Simulated half-lives extracted from eChemPortal for water, soils, and sediments (at reliability levels 1, 2, and 4, www.echemportal.org, accessed May 28, 2020), which were compared to REACH Annex III criteria for P/vP (i.e., >40/>60 days for freshwater; >120/>180 days for freshwater sediment and soil); if a half-life threshold for P or vP was exceeded, the substance from this database would receive that classification herein. (3) Weight-of-evidence persistency conclusions from Berger et al.^126^ or a listing of “broad consensus” of a substances meeting the PBT/vPvB criteria on the ECHA web site’s “advances search for chemicals” (https://echa.europa.eu/advanced-search-for-chemicals, accessed May 31, 2020) to conclude either P or vP. (4) Experimental readily biodegradable screening tests (e.g., OECD301A-F, OECD310) or inherent biodegradation screening tests as extracted from eChemPortal. If all available results concluded “readily/inherently biodegradable”, the substances were classified as “Not P” herein; however, if the number of screening tests reporting “not readily/inherently biodegradable” was equal to or greater than those that did report “readily/inherently biodegradable”, a preliminary conclusion of “Potential P/vP” was assigned. (5) If no other data was available, read-across methods and QSARs were utilized for a weight-of-evidence approach as elaborated below.

The read-across methods were primarily used for per-and-polyfluoroalkylated substances (PFAS) as well as some additional substances in rare cases. Perfluoroalkyl substances are generally considered persistent, and polyfluoroalkylated substances may be persistent or precursors of persistent perfluoroalkyl substances as transformation products.^127,128^ PFAS were identified among the inventory of REACH registered and monitored substances by first filtering substances where the number of fluorine atoms were 50% of the number of carbons or greater. If so, the structure was inspected and classified as a “long-chain” PFAS (having a perfluorinated alkyl chain of 6 carbons or longer), “short-chain PFAS” (having a perfluorinated alkyl-chain of 2–5 carbons), trifluoromethansulfonate (TFMS), trifluoroacetate (TFA), or other highly fluorinated compounds (“other HFCs”). PFAS were considered vP if perfluorinated and “Potential P/vP++” if uncertain. The method of identifying PFAS used here is not consistent with the OECD or EPA definitions and, therefore, would exclude several substances that could be considered PFAS using those definitions.^129,130^

Various QSAR methods were considered and compared for the P/vP assessment. QSARToolbox software (https://qsartoolbox.org/, ver. 4.4, accessed May 28–30, 2020) was used to run EPISuite’s BIOWIN biodegradability QSARS 1 through 6 and the QSARToolbox “P predictor”. The BIOWIN data was processed in two ways. The first was to use the approach in the ECHA PBT/vPvB guideline,^32^ which concludes “Potential P/vP” if the BIOWIN 2 (nonlinear model) or BIOWIN 6 (MITI nonlinear prediction) result is <0.5 and the BIOWIN 3 (ultimate biodegradation time) result is ≤2.25. The other method used was to convert BIOWIN output to estimated half-lives in freshwater using the regression models presented by Arnot et al.,^131^ where the geometric average of all models plus one geometric standard deviation was used to derive an estimated half-life, to err on the side of caution.^41^ The half-life derived using this method is referred to here as the “*t*_1/2_ QSAR”. Another biodegredation half-life QSAR consulted was OPEn structure–activity/property Relationship App (OPERA)^1311^ (accessed via https://comptox.epa.gov/dashboard/batch-search, accessed August 21, 2021). The persistency database produced by ECHA in 2014, and called Pro S.P.,^21^ which provides persistency conclusions (though little traceability) was also consulted.

An approach was developed to see if substances that obtained a “Potential P/vP” assessment based on readily or inherently biodegradability tests could be assessed as P, vP, or “Potential P/vP++” based on weight-of-evidence from QSARs. For this, a comparison of diverse QSAR output with higher quality data (e.g., experimental half-lives or biodegradation tests) was made. The comparison of P/vP conclusions was used to investigate specificity (i.e., persistent substances were correctly predicted as persistent), sensitivity (i.e., not persistent substances correctly predicted as not persistent), and the overall efficiency of all predictions being correct.

#### Mobility Data and Evaluation

Experimental *K*_OC_ and *K*_OW_ data were acquired from two sources. The first was eChemPortal (extracted May 28, 2020), where only experimental or read-across data were extracted at reliability levels 1, 2, and 4. The data was manually curated by removing extremely high values (e.g., >10 log units), due to the suspicion the data was reported incorrectly (e.g., *K*_OC_ values reported as log *K*_OC_ values). The second was the UFZ-LSER database^132^ (accessed September 23, 2020), which provides *K*_OC_ and *K*_OW_ data based on the output of poly parameter free energy relationships (PP-LFER) for neutral substances. These UFZ-LSER outputs are considered of experimental quality if all the PP-LFER descriptors are experimentally determined.^48,133^ For *K*_OC_ data, the PP-LFER of Bronner and Goss^48^ was selected, and for *K*_OW_ it was from Abraham et al.^134^

If multiple log *K*_OC_ values from several studies were given, either the minimum log *K*_OC_ data or the average log *K*_OC_ minus one standard deviation was used for the mobility assessment, to err on the side of caution. A similar consideration was made for experimental values of log *K*_OW_. Many data were reported with the operators <, ≤, ca., >, and ≥. Some of this data had to be excluded as including such operators led to ambiguous mobility conclusions (e.g., a log *K*_OC_ > 1 could be M, vM, or not M). There were frequently occurring log *K*_OC_ entries in eChemPortal of >5.63 or <1.25, which clearly indicate not M or vM, respectively, likely based on the limits of a log *K*_OC_ testing methodology (such as analytical detection limits in the water or soil phase). No discrimination was made in the obtained *K*_OC_ data for pH, temperature or experimental protocol, due to the rarity of such data in the eChemPortal database.

Where *K*_OC_ data was not available, a screening approach was tested using *K*_OW_ and *D*_OW_ data for its reliability in correctly predicting M/vM conclusions based on higher quality *K*_OC_ data. This screening approach was introduced in previous work by the German Environment Agency (UBA), using fewer data points than the current study, which proposed a minimum log *K*_OW_ or minimum log *D*_OW_ < 4.5 could be used as the basis for screening for mobility.^20,21^*EstimatedK*_OC_ values were not considered for the development of a screening or weight-of-evidence approach, despite estimated *K*_OC_ values being available via eChemPortal and the UFZ-LSER database (using estimated PP-LFER descriptors). This was done to be consistent with the PMT/vPvM criteria under discussion to only use the minimum of experimentally measured *K*_OC_ data for this assessment, and because many such methods are calibrated in part with *K*_OW_ data. For this development, estimated *K*_OW_ for neutral species were obtained from two sources: the UFZ-LSER database (by using estimated PP-LFER descriptors instead of the experimental ones) and ChemAxon (accessed September 22, 2019). Minimum *D*_OW_ values between a pH of 4 and 9 were calculated from the data set of best available *K*_OW_ (neutral species) and p*K*_a_ values as follows for all identified acids and bases:
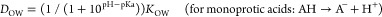
3
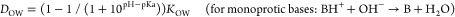
4

Though eqs 3 and 4 are explicitly for monoprotic acids and bases, they were applied to multiprotic acids as well for simplicity, using the p*K*_a_ of the most acidic proton (eq 3) or of the most acidic conjugate acid (eq 4). The minimum *D*_OW_ was calculated for acids at pH 9 and for conjugated acids at pH 4. For amphoteric molecules and zwitterions, which have a complex dependency on pH, the minimum of the eChemPortal data, UFZ-LSER data, or Chemaxon *D*_OW_ predictions between pH 4 and 9 were used as the minimum *D*_OW_ for further analysis. By comparing log *K*_OC_ values with log *K*_OW_ and log *D*_OW_ values for organic compounds that were neutral nonpolar, neutral polar, ionizable and anionic, ionizable and cationic, and zwitterionic, the suitability of the log *K*_OW_ and log *D*_OW_ values of <4.5 as a screening paramater for mobility, or as part of a weight-of-evidence to assess mobility, was tested for each of these polar and ionizability substance classes.

#### Polarity and Ionizability Characterization

All substances were classified as being *neutral nonpolar*, *neutral polar*, *ionizable anionic*, *ionizable cationic*, and *amphoteric/zwitterionic* based on the best available SMILES notation and p*K*_a_ values. As a first point of reference, the presence of a net “+” or “–”charge(s) in the SMILES code of each identified organic constituent when expressed in a non-dative notation (e.g., expressing a nitro group as −N(=O)=O rather than dative bond notation of [N+]([O−])=O), was compiled. A net “+” indicates a cation or a substance that can ionize to a cation; a net “–” indicates an anion or substance that can ionize to an anion; the presence of both “+” and “–” indicates a zwitterion or an amphoteric substance that could ionize to a zwitterion. The best available p*K*_a_ data was taken from the following data sources, in order of priority: experimental p*K*_a_ data from the peer-reviewed literature,^41,135^ experimental p*K*_a_ data values reported in the eChemPortal database available from ECHA and the OECD (https://www.echemportal.org/echemportal/, at reliability levels 1, 2, and 4, accessed May 28, 2020), and finally, if no experimental data was available, estimated p*K*_a_ values using ChemAxon software (https://www.chemaxon.com/, September 22, 2019).

The classification of amphiprotic/zwitterionic was given if the SMILES (in non-dative form) contained both a positive and negative charge (as mentioned above) or alternatively if the structure had both an acidic proton with a p*K*_a_ < 9.3 (i.e., for A–H → A^–^ + H^+^) *and* a conjugate acidic proton with a p*K*_a_ > 3.7 (i.e., for BH^+^ + OH^–^ → B + H_2_O), and therefore would be amphiprotic for the ambient pH range of 4–9. Ionizable anionic or ionizable cationic was used to indicate the substance would either be ionic or transition to an ionic form, within the pH range of 4–9. If the most acidic proton had a p*K*_a_ < 9.3 or the strongest conjugate base had a p*K*_a_ > 3.7, the substance would be classified as transitions to anion (pH 4–9) or transitions to cation (pH 4–9), respectively. As a quality control check, substances that were ionizable anionic and basic or ionizable cationic and acidic were flagged, as this is uncommon. In all cases where this occurred, it was verified to be correct, as these substances would transition from ions to zwitterions depending on pH. As an example, most instances of acidic cations were substances that had a permanently charged cationic group (e.g., a quaternary amine) in addition to an acidic moiety (e.g., a carboxylic acid) elsewhere on the molecule, which allowed them to transition from a cation to a zwitterion with increasing pH. The remaining substances were classified as neutral nonpolar and neutral polar (within the pH range of 4–9), where a polar classification was given if the weight percentage of nitrogen and oxygen in the molecule was greater than 12%.^48,49^

#### Toxicity Data and Evaluation

The toxicity (*T*) assessment used the criteria for toxicity based on Annex VIII of REACH. In summary, these are (i) a long-term no observable effect concentration (NOEC) or effect concentration at 10% (EC10) for marine or freshwater organisms is <0.01 mg/L; (ii) carcinogenic categories 1A or 1B; (iii) germ cell mutagenic categories 1A or 1B; (iv) toxic for reproduction categories 1A, 1B, or 2; and (v) specific target organ toxicity after repeated exposure (STOT RE) categories 1 and 2. Additional categories (Figure 2) were also included due to the additional considerations of long-term exposure to the general population. The additional categories are carcinogenic category 2, cell mutagenic category 2, effects on lactation, a Derived-No-Adverse-Effect-Level (DNEL) for general population (oral, long-term) ≤ 9 μg/kg/day, and endocrine disrupting properties.^20^ NOEC/EC10 data was obtained from the EnviroTox database version 1 (https://envirotoxdatabase.org/, accessed September 7, 2020). Data for the hazard categories, including Endocrine Disruption, were acquired from the ECHA web site’s advanced search for chemicals (https://echa.europa.eu/advanced-search-for-chemicals, accessed May 31, 2020 for harmonized classifications and June 18, 2020 for minority opinions). DNEL data was obtained from the IUCLID 6 database (https://iuclid6.echa.europa.eu/de/reach-study-results, last accessed January 2018). Additional endocrine disruption data was obtained from the CHEMSec SINList of endocrine disrupters (https://sinlist.chemsec.org/, accessed May 30, 2020). Further, a list of suspected endocrine disruptors was obtained from the 2014 Pro S.P.^21^ list mentioned above. If none of the listed toxicity criteria were met, a Cramer Class assessment was conducted using QSAR Toolbox (conducted May 29, 2020), with Cramer Class III being considered “Potential T”. In case a Cramer Class III did not occur, the substance was assumed to be “not T”.

## Results and Discussion

### Monitoring Data Overview

Of the 1289 substances detected in different water media (Table 1), 39% (509 substances) of them were registered under REACH (as of September 2019) as an industrial substance. The remainder consisted of pharmaceuticals, biocides, and agricultural chemicals with no industrial use, and these are therefore not considered under REACH. The proportion of substances monitored in surface water and wastewater included fewer REACH registered substances (38 and 32%, respectively) than those associated with raw water and drinking water (59% and 48%, respectively). The reason for the larger percentage of REACH registered substances detected in drinking water media than surface- and wastewater is not clear. It may be because the surface water and wastewater studies identified in this review tended to be more focused on pharmaceuticals and agricultural chemicals rather than on industrial substances. Alternatively, it may also be that industrial chemicals are used closer to drinking water sources. However, determining whether it is sampling study bias or proximity to drinking water sources that was the explanation for this was not the focus of the current study. Of the 509 REACH registered substances detected in water, 229 of them had registered volumes in Europe of >10 tons per annum, indicating contamination caon occur at low REACH registered tonnages or due to co-contamination from non-REACH uses.

### Polarity and Ionizability

**Figure 3:**
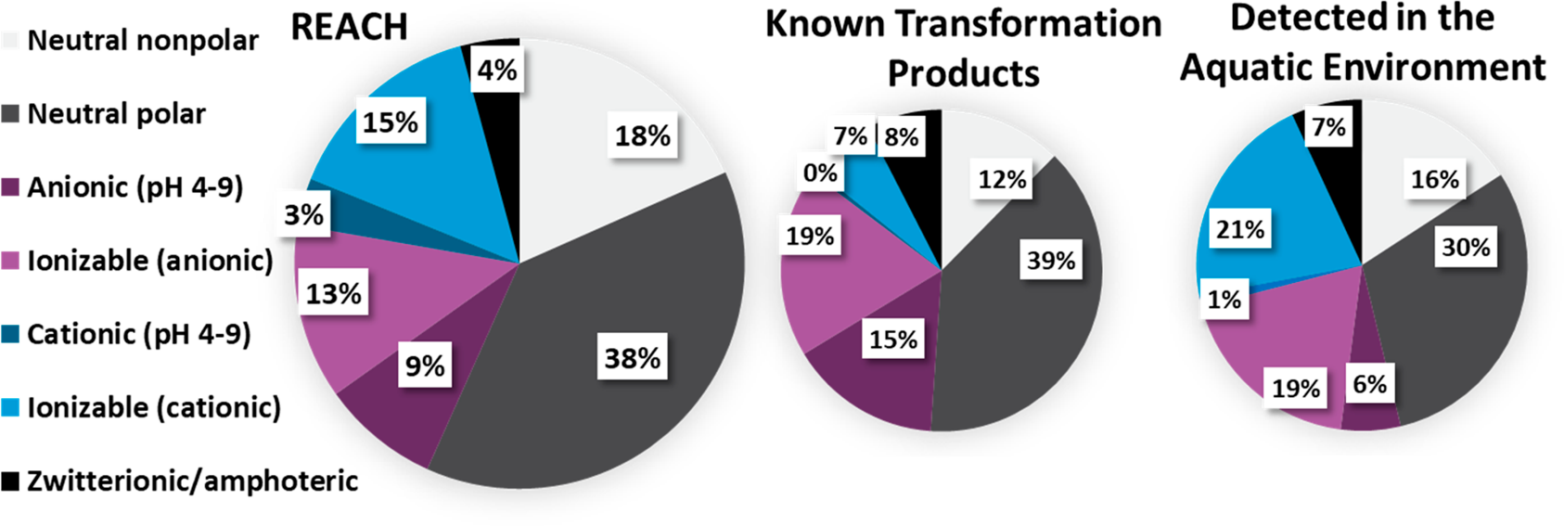
Distribution of polarity and ionizability among identified chemical structures among REACH registered substances (*n* = 12 960), known transformation products of them (*n* = 597), in addition to substances detected in diverse freshwater media (*n* = 1289).

The breakdown of the unique chemical structures identified in the REACH registered substance list (*n* = 12 960), their known transformation products (*n* = 597), and the detected substances (*n* = 1289) were categorized in terms of their polarity and ionizability. The results are show in Table 3 and Figure 3.

**Table 3:** Number of Identified, Unique Chemical Structures among REACH Registered Substances, Identified Transformation Products, and Their Classification Based on Polarity and Ionizability

	REACH-unique organic chemicals	REACH-identified organic transformation products	REACH including trans. products	detected substances in the aquatic environment
all unique structures	12 960	597	13 405	1289
neutral nonpolar	2381	74	2423	204
neutral polar	4970	231	5138	392
anionic (pH 4–9)	1096	91	1179	75
ionizable (transitions anionic)	1629	113	1709	244
cationic (pH 4–9)	438	3	440	14
ionizable (transitions cationic)	1897	40	1924	271
zwitterionic/amphoteric	549	45	592	89

As is evident from Figure 3, the majority of known organic chemicals among REACH registered structures are neutral (56%, with 18% as nonpolar and 38% as polar). This fraction decreases when considering transformation products (51%, with 12% nonpolar and 39% polar), with the fraction of all zwitterion/amphoteric substances increasing (from 4% to 8%), as well as ionizable anions and anions (from 21% to 34%), while the fraction of ionizable cations and cations decreases (from 18% to 7%). This is attributable to oxidative reactions, by either photolysis, aerobic biodegradation, or hydrolysis, often adding polar or negatively charged oxygen moieties (e.g., alcohols, carboxylic acids, etc.).^136^ For the detected substances in freshwater environments, less than half were neutral substances (46%), there was a similar amount of (ionizable) cations (22%) and (ionizable) anions (25%), and 7% were zwitterionic/amphoteric. This may be attributable to ionizable and ionic substances being in general hydrophilic. Overall, it is an interesting though also expected observation that substances detected in water are more likely to be ionizable and ionic compared to REACH registered substances; ionic and ionizable functional groups are an indicator of mobility.

### Persistence

#### Half-Lives and QSARs

Experimental half-life data from simulation tests available from the eChemPortal database were only available for 70 unique substances for freshwater (e.g., using OECD 309), 13 unique substances for marine water (e.g., using OECD 306), 231 unique substances for soil (e.g., using OECD 307), 91 unique substances for freshwater sediments (e.g., using OCED 308), and 3 substances for marine sediments. Considering all media, there were 292 unique substances (2.2%) of the 13 405 REACH and transformation products under consideration where at least one simulated half-life was available. Though this is a much better statistic than the 2013 UNEP report that found that only 220 out of 95 000 substances had half-life data (0.2%), it only further demonstrates that simulated half-lives are extremely rare. This is likely due to the costly nature of the tests required to determine this parameter as well as their complexity and may point to the fact that that weight-of-evidence conclusions of persistency, such as those based on improving persistency QSARs, are needed.

Table 4 shows how well the two QSAR approaches used here for biodegradation half-lives, i.e., *t*_1/2_-QSAR and OPERA, compared with reported experimental half-lives for freshwater, soil and sediment. Maximum experimental half-lives were used for this comparison when there was more than one value available, to err on the side of caution and to account for the fact that some simulated half-life tests may potentially be carried out in the presence of favorable enzymes, catalysts, or conditions that might have resulted in a bias in the data set. The logarithmic difference between maximum simulated half-lives and predicted half-lives, Δlog(*t*_1/2_), was calculated as in eq 6.
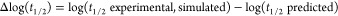
6A positive Δlog(*t*_1/2_) means that the QSAR underpredicted the simulated half-life, and a negative value means that the QSAR overpredicted the simulated half-life. QSAR predictions could not be made for all the substances for which half-lives were available because the chemical structure in question was outside of the application domain of the QSAR models utilized. This was particularly true for organometallic substances or sulfur containing substances (e.g., thiazoles).

**Table 4:** Comparison of QSAR Half-Lives with the Longest Half-Lives Reported from Experimental Simulation Tests Obtained from the eChemPortal Database

	avg Δlog(*t*_1/2_) ± SD	
comparison of simulated vs QSAR half-lives: Δlog(*t*_1/2_) = log(*t*_1/2 experimental, simulated)_- log (*t*_1/2__predicted_)	*t*_1/2_-QSAR	OPERA
experimental maximum: *t*_1/2_ fresh water	–0.5 ± 1.3 *n* = 60)	0.5 ± 1.3 (*n* = 49)
experimental maximum: *t*_1/2_ in soil	–0.5 ± 1.4 (*n* = 221)	0.4 ± 1.5 (*n* = 202)
experimental maximum: *t*_1/2_ in sediment	–0.5 ± 1.2 (*n* = 80)	0.6 ± 1.1 (*n* = 71)

The simulated half-lives were overpredicted by the *t*_1/2_-QSAR output on average by a factor of 3, i.e., Δlog(*t*_1/2_) = −0.5 log units, in all media: water (−0.5 ± 1.3, *n* = 60), soil (−0.5 ± 1.4, *n* = 221), and sediment (−0.5 ± 1.2, *n* = 80). In contrast, the OPERA output tended to under predict half-lives by a factor of 3, i.e., Δlog(*t*_1/2_) = +0.5 log units, in all media: water (0.5 ± 1.3, *n* = 49), soil (0.4 ± 1.5, *n* = 202), and sediment (0.6 ± 1.1, *n* = 71). OPERA predictions were therefore nearly an order of magnitude smaller than *t*_1/2_-QSAR output. As an example, half-lives for PFAS using OPERA were 1–10 days, compared to the *t*_1/2_-QSAR predictions that were 1000–10 000 days. The large standard deviations from both methods, which ranged from 1.1 to 1.5 log units (i.e., a factor 12–30), deserve special attention. When the standard deviation was included, predictions based on *t*_1/2_-QSAR range from underpredicting by nearly a factor of 10 to overpredicting by nearly a factor of 100.

It must be emphasized that this comparison did not manually investigate the accuracy or appropriateness of all half-life data from the eChemPortal database, as the purpose was not to develop or calibrate QSARs. Instead, Table 4 shows how a filtered data set of experimental half-lives from eChemPortal compares with QSAR predictions. Simulated half-lives can vary across the literature from sources other than eChemPortal. For instance, the maximum half-life for hexabromocyclododecane in sediments was reported as 32 days on eChemPortal, whereas a half-life of 191 days was reported in the peer reviewed literature.^137^ Some simulated half-life data may be obtained under conditions that are favorable to degradation, such as in studies developing a remediation technology, where a catalyst or specific enzymes may be present, e.g., for carbon tetrachloride.^138^ Both the *t*_1/2_-QSAR and OPERA models could in principle be further calibrated based on new half-life data that has become available since these models were last calibrated. However, this was not the focus of the current study but is very much worth looking into in the future.

The large standard deviations that are obtained when using both the *t*_1/2_-QSAR and OPERA half-life predictions indicate that these models are not suitable to be used on their own for half-life predictions that will be used in risk assessment. Nevertheless, they may have a role as part of a weight-of-evidence P/vP hazard assessment in combination with other data, so long as their uncertainty is taken into consideration. The number of times *t*_1/2_-QSAR predictions, OPERA predictions, as well as the QSARToolbox P profiler output gave a conclusion of P in water, soil, or sediment, or alternatively “Not P” in all three media, that agreed with the available simulated half-life data was compiled. The results are presented in Table 5. For this purpose, an estimated half-life of ≥40 days was set as the cut-off for persistence based on the REACH Annex XIII definition of persistence in water. Table 5 shows that *t*_1/2_-QSAR predictions ≥40 days and the QSARToolbox P profiler predictions matched the available persistency conclusions from simulation tests for 74% (*n* = 78) and 78% (*n* = 55) of applicable substances, respectively. OPERA, however, only agreed with this conclusion 19% (*n* = 72) of the time, as it tended to under predict reported half-lives. The predictions from *t*_1/2_-QSAR and QSARToolbox, agreed with each other in most instances.

**Table 5:** Comparison of QSAR Conclusions of Persistency with Those of Reported Simulated Half-Lives in Water, Soil, and Sediment and the REACH Annex XIII Criteria for Persistence (P) And Very Persistent (vP)^a^

comparison of QSAR conclusions with simulation test half-life conclusions		QSAR max *t*_1/2_	*n*
*t*_1/2_-QSAR	P in water, soil **OR** sediment agrees with *t*_1/2_-QSAR ≥ 40 days	74%	78
	”Not P” in water, soil **AND** sediment agrees with *t*_1/2_-QSAR < 40 days	40%	5
	*overall efficiency*	72%	83
OPERA	P in water, soil, **OR** sediment agrees with OPERA ≥ 40 days	19%	72
	”Not P” in water, soil, **AND** sediment agrees with OPERA < 40 days	100%	3
	*overall efficiency*	23%	75
QSARToolbox	P in water, soil, **OR** sediment agrees with QSARToolbox profiler	78%	55
	“Not P” in water, soil, **AND** sediment agrees with the QSARToolbox profiler	50%	4
	*overall efficiency*	76%	59

a There are fewer predictions for not Persistent (Not P) as this comparison was required for simulated half-lives in all water, soil, and sediment media. Overall efficiency refers to the frequency of times P and “Not P” were predicted correctly.

#### Screening Tests and QSARs

Readily biodegradable screening tests (e.g., OECD301A-F, OECD310) or inherently biodegradable screening tests were available for 3740 substances, of which 2216 chemicals were concluded as “Not P” and the remaining 1524 as “Potential P/vP”. Table 6 compares QSAR predictions to the results of the screening tests, using the assumption that an output of ≥28 days would be “Potential P/vP” and <28 days “Not P” (28 days was chosen as the threshold, as it is typically used in OECD301 and 310 tests). The substances with a *t*_1/2_-QSAR output of ≥28 days matched for 80% of the substances where “Potential P/vP” was concluded from the readily/inherently biodegradable screening tests (*n* = 1365). But among the substances with a *t*_1/2_-QSAR output of <28 days, only 68% had a “Not P” conclusion based on these screening tests (*n* = 2159), giving an overall efficiency of 73% (*n* = 3524). By contrast, using the 28 day cutoff, OPERA was better at predicting “Not P” as 95% of the “Not P” substances (*n* = 1747) were predicted correctly. However, due to the general underestimations of half-lives (Table 5) exhibited by OPERA, it was extremely poor at predicting “Potential P/vP” with only 16% of predictions being correct. Overall, the efficiency of OPERA was 65% (*n* = 2818). The predictions of “Potential P/vP” with the ECHA recommended BIOWIN method^32^ matched for only 34% of the substances where “Potential P/vP” was concluded from the readily/inherently biodegradable screening tests. This is a much lower specificity than the *t*_1/2_-QSAR output of ≥28 days; the sensitivity of the ECHA recommended BIOWIN method was not tested, as this method was not developed for the screening of “Not P”.

**Table 6:** Comparison of QSAR Conclusions with Those of Readily/Inherently Biodegradable Screening Tests^a^

comparison of readily/inherently biodegradable tests (compiled) with various QSARs		QSAR maximum *t*_1/2_ (d)	*n*
*t*_1/2_-QSAR	not readily/inherently biodegradable **AND***t*_1/2_-QSAR ≥ 28 days	80%	1365
	readily/inherently biodegradable **AND***t*_1/2_-QSAR < 28 days	68%	2159
	*overall efficiency*	73%	3524
OPERA	not readily/inherently biodegradable **AND** OPERA ≥ 28 days	16%	1071
	readily/inherently biodegradable **AND** OPERA < 28 days	95%	1747
	*overall efficiency*	65%	2818
BIOWIN-ECHA	not readily/inherently biodegradable agrees with the BIOWIN-ECHA PBT Guideline^32^ method for Potential P/vP	34%	1401

a Overall efficiency refers to the frequency at which “Potential P/vP” and “Not P” were predicted correctly.

Given the uncertainty in the *t*_1/2_-QSAR predictions that showed that half-lives can be underpredicted by a factor 10, a *t*_1/2_-QSAR cutoff of 400 days was used to see if this value was suitable to identify “Not P” substances. Here, 400 days was chosen because it corresponded to a factor of 10 greater than the REACH half-life threshold for water of 40 days. Only 0.4% of the confirmed “Not P” substances based on laboratory experiments had a predicted average *t*_1/2_-QSAR above 400 days (or 54 out of 2200 “Not P” substances, i.e., a sensitivity of 99.6%); of these, 12 had structural flags and the remainder had large molecular weights (260–1300 Da), with some capable of hydrolysis (e.g., 6-PPD, CAS 793-24-8). Therefore, the lack of a 100% match may be due to (a) the applicability domain for the *t*_1/2_-QSAR not being applicable for larger substances and (b) hydrolysis not being considered. Therefore *t*_1/2_-QSAR cutoffs of >400 days may be a suitable parameter to conclude “Potential P/vP” or, as part of weight-of-evidence, to conclude P or vP, particularly if hydrolysis can be ruled out.

#### Persistence Assessments

**Figure 4:**
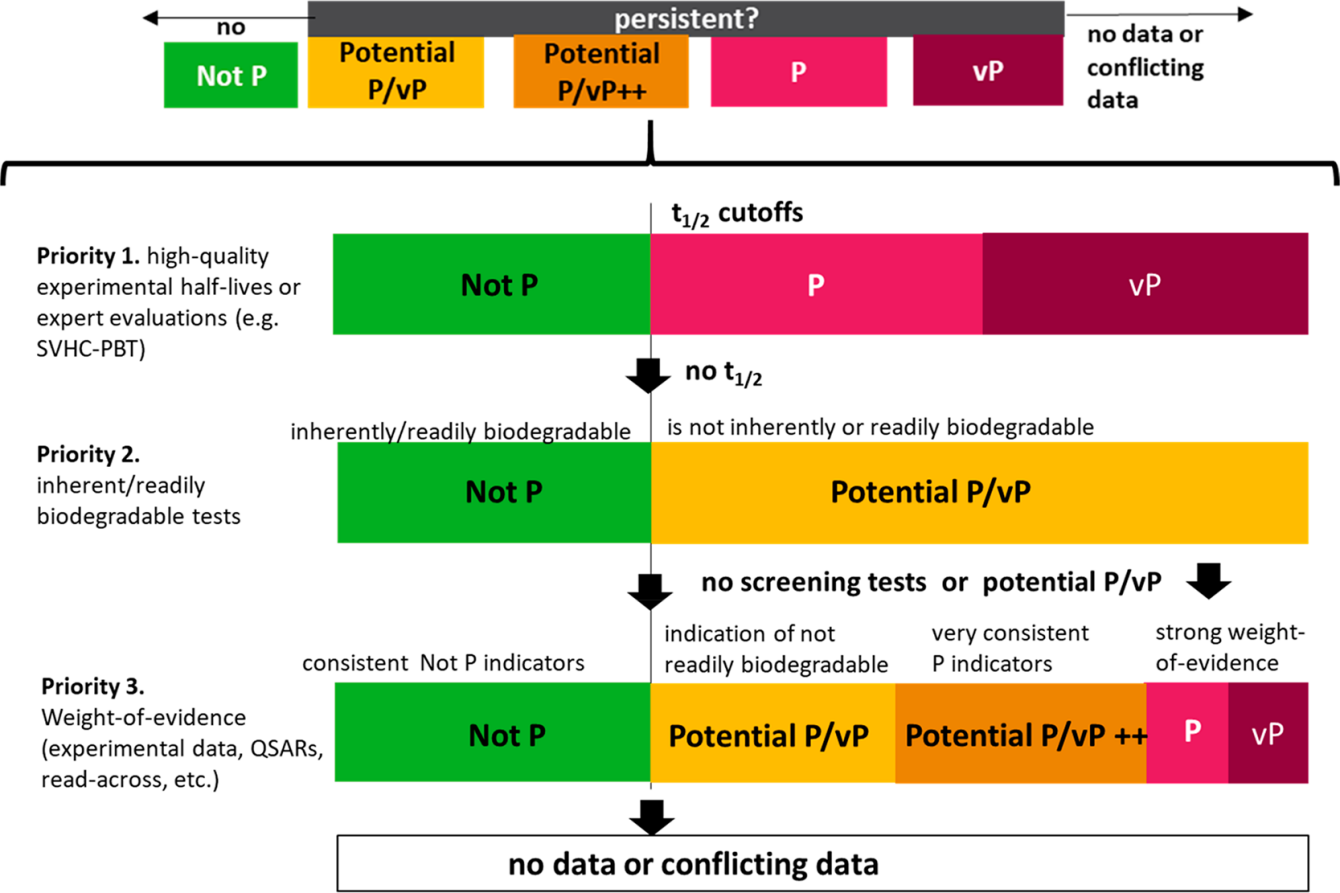
Three tiered priority levels of conducting a P/vP assessment as part of the PMT/vPvM assessment presented in Figure 2. The Priority 1 tier is based on high-quality simulated half-lives, *t*_1/2_, compared to the relevant thresholds, or expert evaluations if available. The Priority 2 tier is based on inherent or readily biodegradable screening tests that can be used to screen for “Not P” or “Potential P/vP”. If no screening tests are available or the conclusion of them was “Potential P/vP”, then Priority 3 assessments are made using diverse weight-of-evidence indicators, including screening tests, QSARs, experience with removal during drinking water purification, and other evidence. SVHC-PBT = substances of very high concern because of its persistent, bioaccumulative and toxic properties or very persistent, very bioaccumulative properties as defined in the REACH regulation.

Figure 4 presents three priority levels or tiers to use when approaching P/vP assessments. These tiers are consistent with REACH Annex XIII.^32^ In Figure 4, a “Priority 1” P/vP assessment is based on either high-quality simulated half-lives or harmonized P/vP assessments based on the REACH criteria. “Priority 2” assessments are based on readily/inherently biodegradability tests that allow for either a conclusion of “Not P” or “Potentially P/vP”. Finally, the “Priority 3” assessment is based on additional weight-of-evidence assessment to the readily/inherently biodegradable test data, obtained from the use of QSARs or other data, to make an assessment on a case-by-case basis.

For the “Priority 3” weight-of-evidence persistency assessment, first literature data were consulted if available. If no previous weight-of-evidence persistency assessment was available in the literature, a decision tree was utilized based on the QSAR data tested in this study. The use of the decision tree depended on whether there was “Priority 2” readily/inherently biodegradability test data available and whether they resulted in the conclusion “Potential P/vP” (Figure 4). If there was no “Priority 2” readily/inherently biodegradability screening tests available, a substance was considered:

(i) “Not P” if data from *all* QSARs tested here indicated “Not P” (including OPERA, “Pro S.P.”, QSARToolbox, and a *t*_1/2_-QSAR half-life <28 days);
(ii) “Potential P/vP” if data from all QSARs excluding OPERA gave consistent conclusions of P/vP OR the substance was detected in drinking water sources, to err on the side of caution;
(iii) “Potential P/vP++” based on additional weight-of-evidence on a case-by-case basis (e.g., known to be difficult to removal during water treatment, ubiquity in monitoring data, read-across in the case of PFAS);
(iv) “No data/low quality data” if the substance was outside the domain of QSARs or if the QSARs gave a conflicting result if the substance was “Not P” or “Potential P/vP”.
  If the conclusion from the “Priority 2” readily/inherently biodegradability test was “Potential P/vP”, then at the “Priority 3” level a substance was considered to be something other than "Potential P/vP" if any of the following applied:
(v) “Not P” if additional evidence existed on a case-by-case basis to conclude this, such as if the substance is rapidly hydrolyzable under ambient conditions^32^ (as an example: 6-PPD, CAS 793-24-8, is not readily biodegradable, but readily hydrolyzable^139^);
(vi) "Potential P/vP++", "P" or "vP" if all the QSARs (excluding OPERA) gave output and concluded persistence (for “P” or “vP” this additionally requires that the *t*_1/2_-QSAR was greater than 400 and 600 days, respectively), and a literature review for each case found no reason to conclude otherwise.
(vii) Case-by-case information to conclude “Potential P/vP++”, “P”, or “vP” based on additional information (e.g., drinking water ubiquity, difficulty to remove from drinking water production, read-across in the case of PFAS, etc).

**Figure 5:**
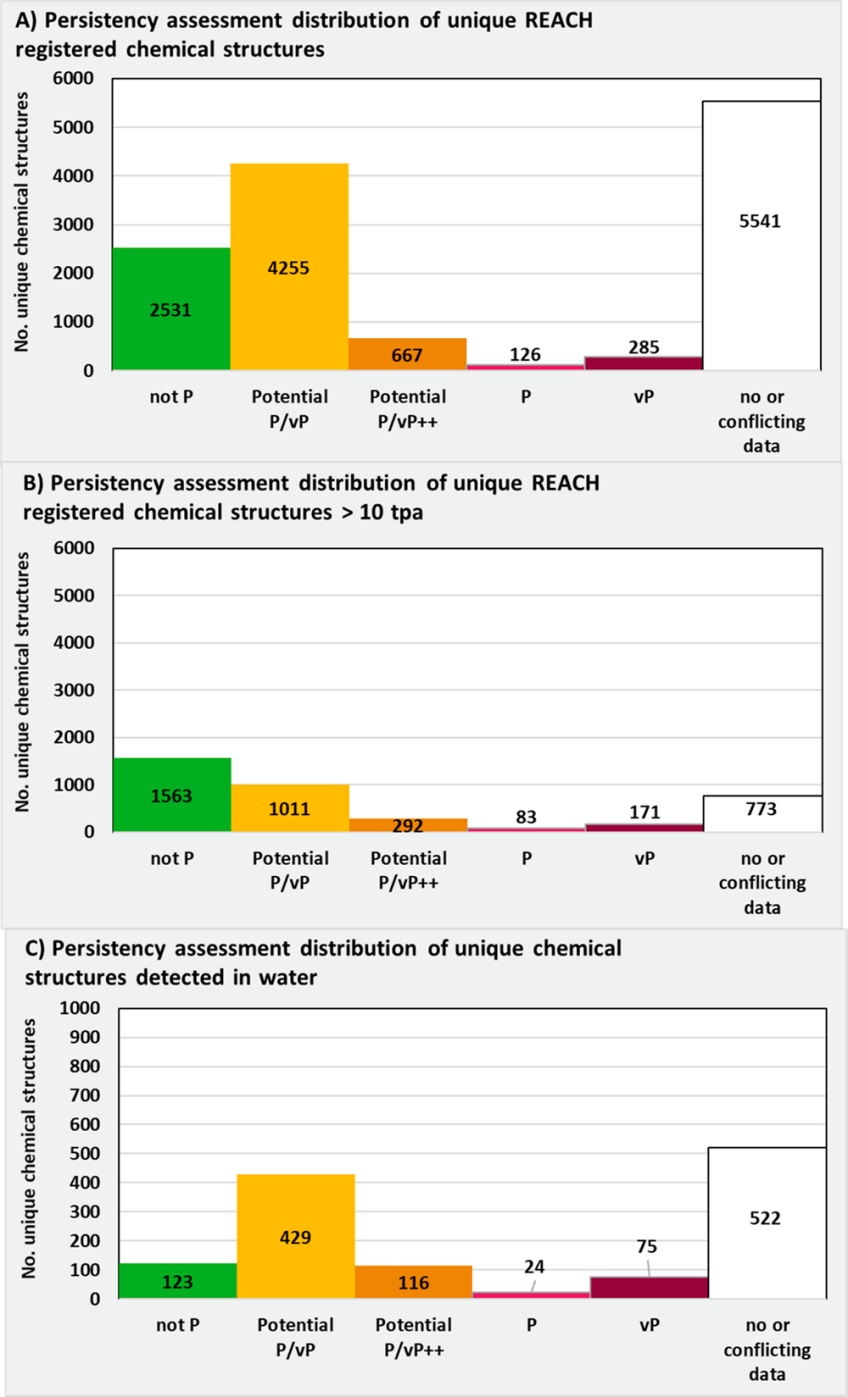
Overview of persistency conclusions for (A) all unique chemical structures identified among REACH registered substances and their transformation products (*n* = 13 405), (B) specifically those registered at volumes of 10 tonnes per year or greater (*n* = 3891), and (C) unique chemical structures detected in water monitoring studies (*n* = 1289)

In Table S1, the results of the persistency assessment are presented for the 14203 substances considered in this study based on the presented workflow (Figure 2, Figure 4). A summary of this persistency assessment is presented for all REACH registered substances and detected substances in water resources in Figure 5.

As is evident from Figure 5, there was a large portion of unique chemical structures registered under REACH where there was insufficient data to make a persistency assessment (41% of structures, *n* = 5541). This was due either to a lack of data or only conflicting data being available (following the Priority 3 assessment described above). Similarly, for the unique chemical structures detected in the literature monitoring studies, there was insufficient data to make a persistency assessment, due to no data or only conflicting data being available for 41% of chemical structures (*n* = 522). However, for unique chemical structures registered under REACH with volumes of over 10 tons per annum (Figure 5B), there is much more data available, with information being available for all but 20% of the substances (*n* = 773). This is attributable to a persistent, bioaccumulative and toxic/very persistent and very bioaccumulative (PBT/vPvB) assessment being required for substances with tonnages > 10 tons per annum based on Article 14 of the REACH regulation. A substantial percentage of substances in each group were given the uncertain conclusion of “Potential P/vP”. This comprised 32%, 26%, and 35% of the identified unique chemicals registered under REACH, those registered at volumes greater than 10 tons per annum, and detected substances, respectively. Considering the “Potential P/vP” and “No or conflicting data” categories together, there is evidently an extremely large data gap in understanding the persistency of substances in the environment, as has been highlighted several times previously in the literature.^41,140,141^

Conclusions of “Not P” occurred for 19%, 40%, and 9% of substances falling in the categories of REACH registered substances, those produced above 10 tons per annum, and those detected in freshwater, respectively. The proportion of substances considered P/vP were 3.1%, 6.5%, and 7.7%, respectively (or if Potential P/vP++ is included then 8.0%, 14.0%, and 16.7%, respectively). In total, there were 460 substances considered P or vP, with the primary reasons for this conclusion being either (i) existing ECHA classification of P/P (48 substances); (ii) simulated half-lives compiled in this study (69 substances); (iii) inclusion of a PFAS moiety (59 substances); (iv) manual weight-of-evidence conclusions in this or other studies in the literature (284 substances).

Several biodegradable, “Not P” substances are detected in water monitoring studies (Figure 5C). P/vP assessments alone are not able to predict whether a substance will be detected in water monitoring studies. Other factors beyond persistence play a role regarding whether substances are detected in drinking water or other media. These factors include mobility, emission rates, emission pathways, and also the heterogeneity of real-world degradation half-lives themselves.^139,142^

### Mobility Data

As with the previous section on the availability and comparability of persistence data of varying levels of quality, this section focuses on the availability and comparability of mobility data, specifically of *K*_OC_ data for the mobility threshold and *K*_OW_ and *D*_OW_ data as screening parameters.

#### Experimental *K*_OC_ Data

Table 7 presents a comparison of the experimental log *K*_OC_ data from the eChemPortal database with the values derived from the UFZ-LSER database using experimental PP-LFER descriptors.

**Table 7:** Comparison between Experimental *K*_OC_ Data from the eChemPortal Database and Those from UFZ-LSER Database Determined with Experimental PP-LFER Descriptors

substance class	Δlog *K*_OC_ = log *K*_OC_ (experimental) – log *K*_OC_ (UFZ-LSER)	*n*
neutral nonpolar	0.0 ± 0.6	102
neutral polar	0.5 ± 0.8	111
ionizable, transition to a cation	1.8 ± 1.8	32
ionizable, transitions to an anion	0.2 ± 1.4	22

From Table 7, the comparisons of log *K*_OC_ data from eChemPortal and UFZ-LSER were best for neutral nonpolar substances, with an agreement of a factor 4 (or 0.6 log units). For neutral polar substances, the experimental values were higher than the UFZ-LSER database values by on average a factor of 3, with a standard deviation of a factor 6. For ionizable substances that transition to an anion (within a pH 4–9), there was on average a good agreement, but the standard deviation was large (factor of 25, or 1.4 log units). For ionizable substances, that transition to a cation (within the pH range of 4–9), the experimental values were substantially larger than the values from the UFZ-LSER database, by on average a factor of 100 with a standard deviation of a factor 100. These discrepancies can largely be accounted for by the UFZ-LSER database mainly being developed for neutral substances and the neutral form of ionizable substances.^132^ The larger experimental *K*_OC_ values for the ionizable substances that transition to cations than the UFZ-LSER prediction is due to the expected extra ionic-exchange interactions with organic matter or minerals in the soil, which tend to have a substantial cation exchange capacity.^60,72,143^ Similarly, the large standard deviation for ionizable substances that transition to anions is due to a broad range of ion exchange and potentially ion repulsion interactions.^70^ Anions are known to exhibit a broad range of experimental log *K*_OC_ values; for instance, the range of log K_oc_ values for PFOS and PFOA are from 2.4 to 4.4 and from 1.3 to 4.5, respectively.^144^ The deviation for neutral, polar substances between eChemPortal and UFZ-LSER was unexpected, as the database has previously performed well for these substances.^48,49,133,145^ The discrepancy here may be due to poor quality experimental log *K*_OC_ values in the eChemPortal database, as these were not checked individually for quality but rather accepted as is, unlike previous comparisons of experimental *K*_OC_ values with LSER descriptions.^48,49,133,145^ Therefore, the UFZ-LSER predictions based on quality-controlled experimental descriptors are considered of higher quality than the eChemPortal data.

#### p*K*_a_ Data and QSARs

Table 8 compares experimental p*K*_a_ data^41,135^ to estimations from Chemaxon, specifically considering the most acidic proton of the substance or conjugate acid. In general, p*K*_a_ predictions match the best for substances with a single acidic proton (either acids or conjugate acids), with an average deviation of 0.1 ± 1.1 log units (*n* = 166). The worst agreement was for the p*K*_a_ of amphoteric substances, where the agreement was 0.9 ± 3.2 (n = 265). This is attributable to the inherent complexity of their pH dependent ionization behavior and indicates speciation predictions are the most uncertain for these substances.

**Table 8:** Comparison of Experimental p*K*_a_ Values of Most Acidic Proton and Those Predicted by ChemAxon

Ionization class	Δp*K*_a_ = p*K*_a_ (experimental) – p*K*_a_ (Chemaxon)	*n*
all ionizable substances	0.5 ± 2.6	521
just one proton (acid or conjugate acid)	0.1 ± 1.1	166
acids (mono and multiprotic)	0.3 ± 1.9	89
bases (mono and multiprotic)	0.0 ± 1.2	167
amphoteric substances	0.9 ± 3.2	265

#### Mobility Data Distribution

**Figure 6:**
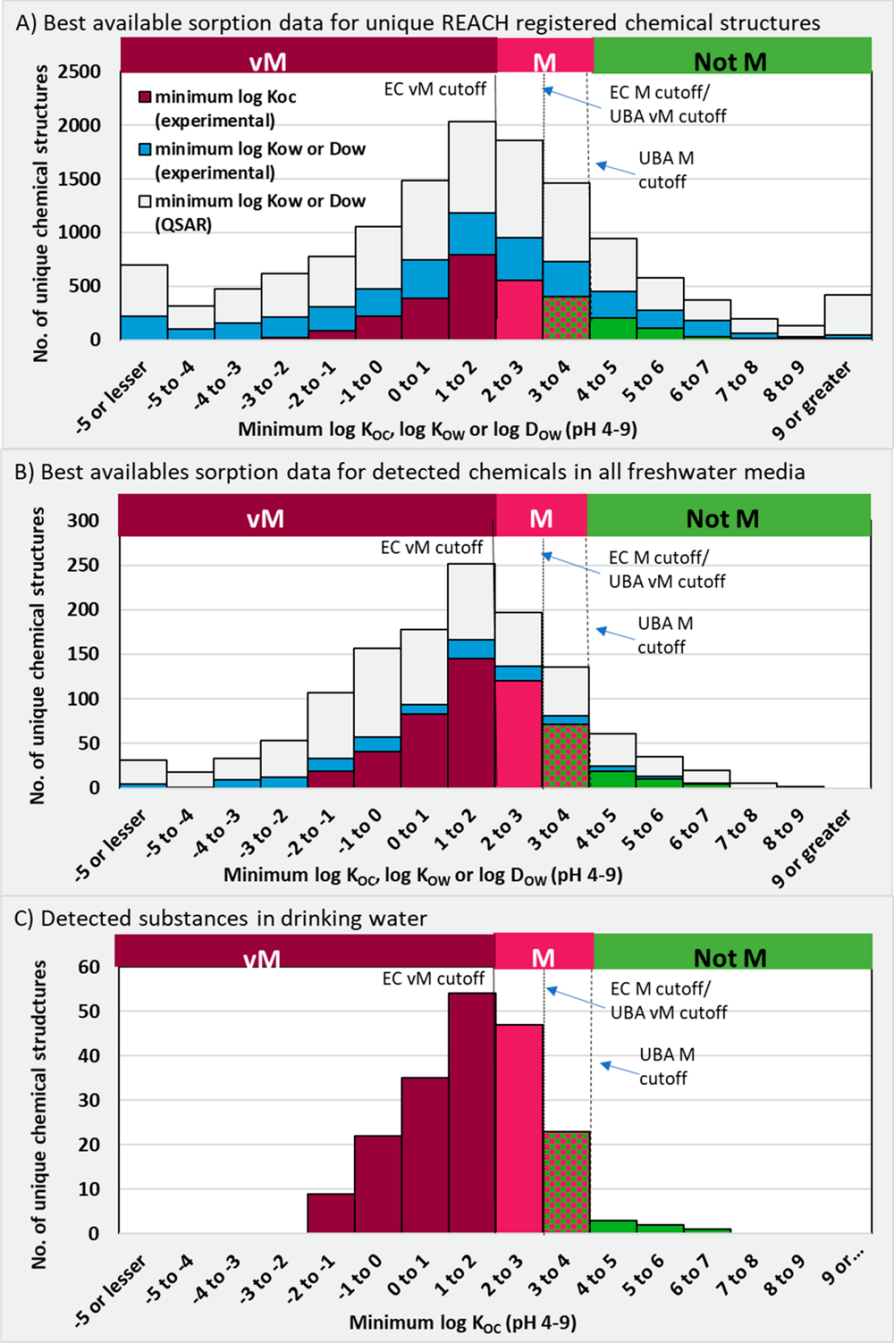
Distribution of best available sorption data for unique chemicals identified in (A) the REACH registered list of substances and (B) detected chemicals in freshwater environments. Also presented is (C) minimum experimental log *K*_OC_ values for substances detected in drinking water (*n* = 196). Also presented is the UBA’s M and vM thresholds (cutoffs) proposed in 2019 at log *K*_OC_ 4.0 and 3.0, respectively, as well as the EC proposed M and vM thresholds (cutoffs) proposed in 2021 at log *K*_OC_ 3.0 and 2.0, respectively. Experimental log *K*_OC_ values are shown in different colors based on their relation to these thresholds (dark fuchsia and fuchsia = vM and M, respectively, according to the EC proposed criteria; tiled = M according the UBA proposed criteria, not M according to the EC proposed criteria only, green = Not M).

Among the 14 203 unique organic chemicals considered in this study, it was possible to obtain experimental *K*_OC_ values for 3072 of them, with 1572 coming from eChemPortal, 1470 from UFZ-LSER database, and 30 from additional literature sources (see the Supporting Information). For the remaining substances, a minimum log *K*_OW_/log *D*_OW_ was available that was either experimentally determined (*n* = 3183) or estimated (n = 7810). Figure 6 shows a histogram distribution of the best available sorption coefficient (where the best is experimental log *K*_OC_, the second best is the minimum experimental log *K*_OW_/log *D*_OW_ (pH 4–9), and the worst is the estimated log *K*_OW_/log *D*_OW_ (pH 4–9)) for all unique, identifiable organic chemicals in the REACH registration database (Figure 6A) and detected substances from the literature studies (Figure 6B). The minimum experimental log *K*_OC_ is considered the best available data, followed by the minimum experimental log *K*_OW_/log *D*_OW_ (*n* = 3262) and the estimated log *K*_OW_/log *D*_OW_ (*n* = 7858) of the lowest priority. No mobility descriptor could be estimated for 11 substances (mainly organometallics, for which none of the QSARs gave output). Several interesting trends can be seen from the histograms in Figure 6, such as the following: (1) the peak frequency of both log *K*_OC_ and log *K*_OW_/log *D*_OW_ is between log 1.0 to log 2.0, implying that this is the most common range of these sorption descriptors for organic substances registered under REACH and detected in freshwater; (2) most chemicals registered under REACH and detected in the environment have either a log *K*_OC_ < 4.0 (87% and 94%, respectively) or a log *K*_OW_/log *D*_OW_ < 4.5 (79% and 88%, respectively); and (3) *K*_OC_ data is more commonly available for environmentally detected substances than REACH registered substances, likely due to more sorption studies being available for detected substances.

Figure 6C presents a histogram of the minimum experimental log *K*_OC_ values for substances detected in drinking water, compiled in this study. The histogram shows that the clear majority of detected substances have a log *K*_OC_ < 4 (190 out of 196, or 97% of substances); this observation is consistent with the UBA proposed threshold for the M criterion. A substantial number of substances also have a log *K*_OC_ < 3, which corresponds to the UBA proposed vM threshold and the M threshold proposed by the EC (167 out of 196, or 90% of substances).^20,21^

Even though most organic substances considered here have a log *K*_OC_ < 4.0, including those in drinking water, they would not be considered as PMT/vPvM substances unless they also meet the P/vP criteria. To present this data, Table 9 contains the distribution of log *K*_OC_ and log *D*_OW_/*K*_OW_ data for all REACH registered substances as well as detected substances that were assessed as P/vP and Potential P/vP++. For the REACH registered substances assessed as persistent with a measured log *K*_OC_ available (*n* = 419), 81% and 64% have a log *K*_OC_ of <4.0 and <3.0, respectively. The percentages of persistent, detected substances with a log *K*_OC_ < 4.0 were larger than that of the persistent, REACH registered substances, including for wastewater effluent (97%), surface water (84%), bank filtrate (100%), groundwater (94%), raw water (96%), and drinking water (98%); this also applied to persistent, detected substances with a log *K*_OC_ < 3.0, including for wastewater effluent (76%), surface water (67%), bank filtrate (92%), groundwater (86%), raw water (85%), and drinking water (82%). Therefore, the persistent substances detected in all water media were more likely to have a log *K*_OC_ of <3.0 or <4.0 than REACH registered substances, except for surface water which had similar percentages. The distribution of log *K*_OC_ values in Table 9 and Figure 6C collectively show how diverse soil, sediment, and sludge media have the ability to partially remove substances with high log *K*_OC_ values due to sorption processes being operational, as the proportion of substances detected with a log *K*_OC_ > 4.0 are very small (<4%) for wastewater, bank filtrate, groundwater, raw water, and drinking water. This data provides justification that the combination of t_1/2_ and log K_OC_ are fit-for-purpose for PMT/vPvM assessment.

**Table 9:**
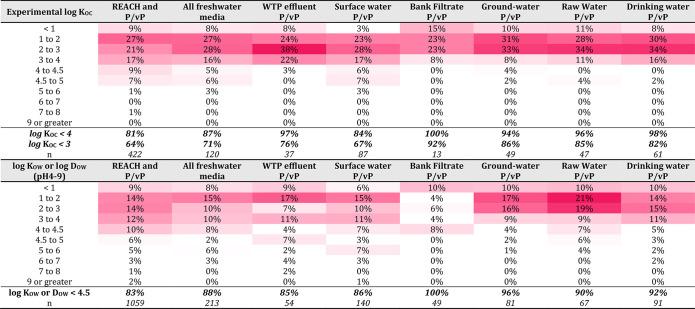
Percentage of Unique Chemical Structures Assessed as Persistent (P), Very Persistent (vP) or Potential P/vP++, among REACH Registered Substances and Detected in Different Water Media That Fall within Specified log *K*_OC_ or Log *K*_OW_/*D*_OW_ Ranges^a^

a The darker the shading, the larger the percentage of chemical structures that fall within a specified *K*_OC_ or log *K*_OW_/*D*_OW_ range. Also shown is the percentage of P, vP, and Potential P/vP++ substances with a log *K*_OC_ of <4, <3 or a log *K*_OW_/*D*_OW_ of <4.5.

#### *K*_OC_ and Screening Descriptors *K*_OW_ and *D*_OW_

**Figure 7:**
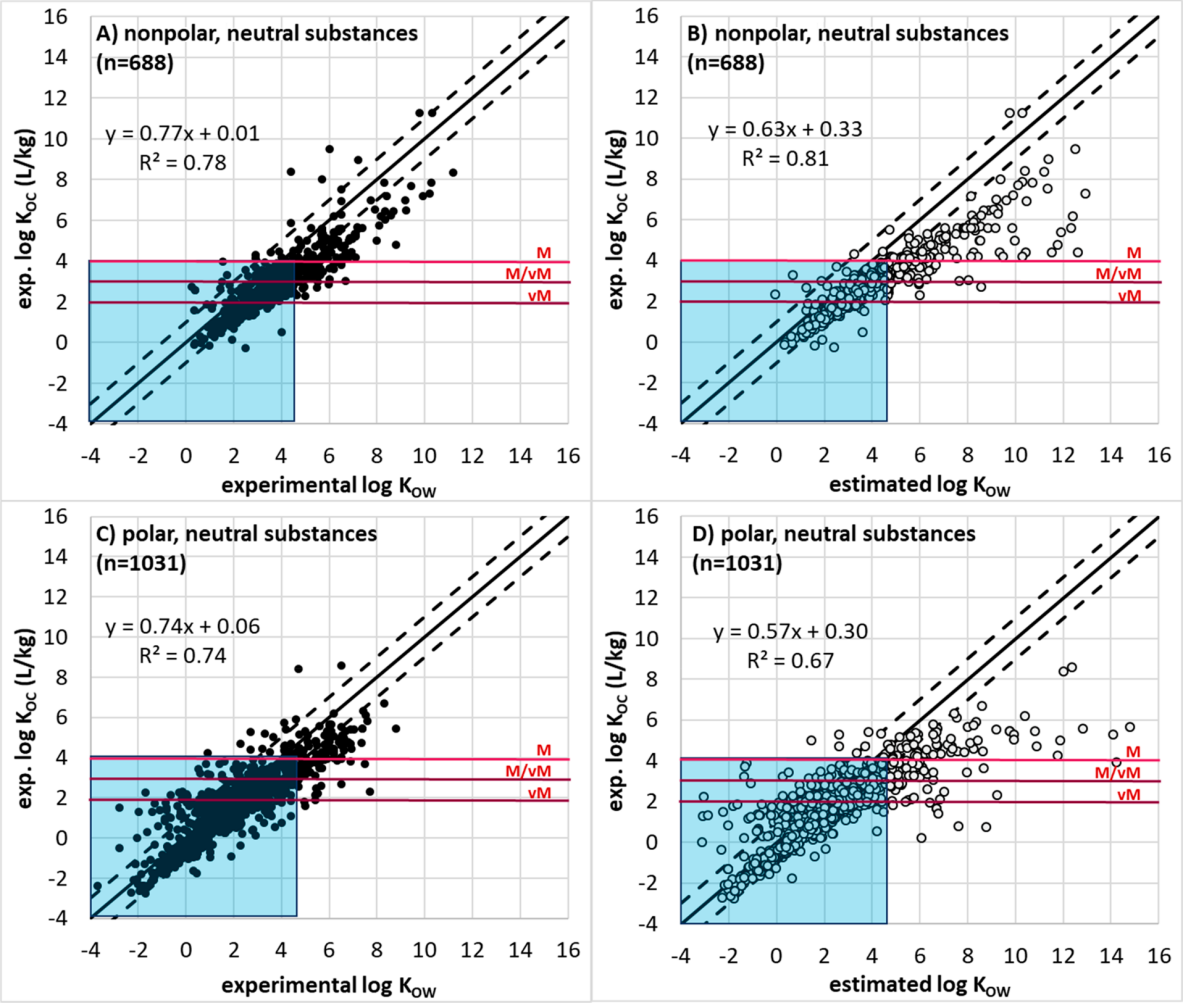
Experimental log *K*_OC_–log *K*_OW_ plots for neutral substances, showing (A) nonpolar substances and experimental log *K*_OW_ values, (B) nonpolar substances and estimated log *K*_OW_ values, (C) polar substances and experimental log *K*_OW_ values, and (D) polar substances and estimated log *K*_OW_ values. Here polar substances are considered those with a mass fraction of oxygen and nitrogen being 12% or above of the molecular mass. The solid line indicates the 1:1 line, with the two dotted lines showing deviations of a factor 10. Shaded blue areas indicate the area with a log *K*_OC_ < 4.0 and log *K*_OW_ < 4.5 to visually illustrate the proportion of substances meeting both the *K*_OC_ criteria and *K*_OW_ screening criteria the UBA proposed in 2019.^20^ Also presented in red lines are the current log *K*_OC_ based mobile and very mobile criteria the UBA proposed in 2019, with thresholds at <4.0 and <3.0, and the EC proposed in 2021, with thresholds at <3.0 and <2.0.

**Figure 8:**
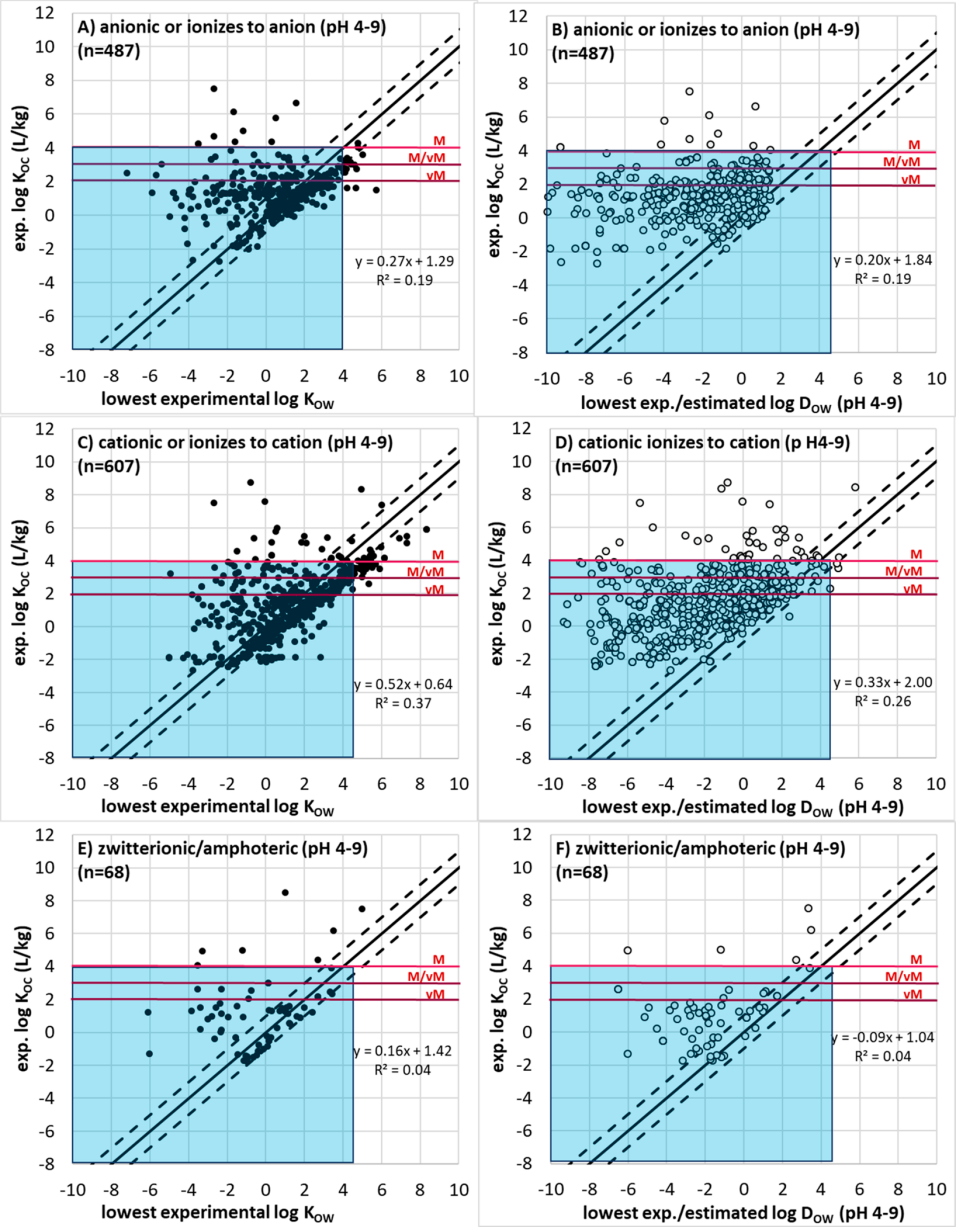
Experimental log *K*_OC_–log *K*_OW_/*D*_OW_ plots for ionic and ionizable substances, with panels (A), (C), and (E) showing comparisons with the experimental log *K*_OW_ values of the neutral species for ionizable anionic, ionizable cationic and zwitterionic/amphoteric substances, respectively, and panels (B), (D), and (F) showing comparisons with the lowest log *D*_OW_ between pH 4 and 9 for ionizable anionic, ionizable cationic and zwitterionic/amphoteric substances, respectively. The solid line indicates the 1:1 line, with the two dotted lines showing deviations of a factor 10. Shaded blue areas indicate the area with a log *K*_OC_ < 4.0 and log *K*_OW_ < 4.5 to visually illustrate the proportion of substances meeting both the *K*_OC_ criteria and *K*_OW_ screening criteria set by the UBA proposed in 2019.^20^ Also presented in red lines are the current log *K*_OC_ based mobile and very mobile criteria the UBA proposed in 2019, with thresholds at <4.0 and <3.0, and the EC proposed in 2021, with thresholds at <3.0 and <2.0.

Experimental *K*_OC_ data for the mobility assessment was available for approximately 20% of the 14 203 substances considered in this study, which is far higher than the simulated half-life data for the persistency assessment which was only available for 2.2% of substances. As presented in the Introduction, *K*_OW_ and *D*_OW_ are often used as proxies for *K*_OC_ values, despite these parameters not accounting for specific polar or ionic interactions with soil organic carbon other components like minerals.^51,61,143,146^ Nevertheless, from a screening point of view, *K*_OW_/*D*_OW_ do not need to be exact *K*_OC_ proxies, as the goal of a screening parameter would be to screen for candidates that are suspected to be mobile substances. The suitability of *K*_OW_/*D*_OW_ as screening parameters in cases where mobility is likely or suspected (i.e., a “Potential M/vM” substances) was therefore investigated. For this purpose, a correlation analysis was carried out for substances that had experimental log *K*_OC_, experimental *K*_OW_, and estimated *K*_OW_/*D*_OW_ parameters available. The following were plotted: experimental log *K*_OC_ data was plotted against log *K*_OW_ for neutral nonpolar substances (*n* = 689), neutral polar substances (*n* = 1032), substances that are anionic or ionize to an anion (*n* = 487), substances that are cationic or ionize to a cation (*n* = 607), and zwitterions/amphoteric substances, as defined by their structure (*n* = 71). These plots are presented in Figure 7 for neutral substances and Figure 8 for ionic/ionizable substances, with regression statistics presented in Table 10.

**Table 10:** Comparison of Experimental Log *K*_OC_ Values with Experimental and Estimated Log *K*_OW_ Values as well as Minimum Log *D*_OW_ Values (pH 4–9) for Neutral and Ionizable Substances^a^

		M Substances	Not M substances				
chemical category		log *K*_OC_ < 4, log *K*_OW_/*D*_OW_ < 4.5	log *K*_OC_ ≥ 4, log *K*_OW_/*D*_OW_ ≥ 4.5	overall efficiency	linear regression	*r*^2^	rmse
neutral nonpolar	exp. log *K*_OW_	88%	91%	89%	log *K*_OC_ = (0.77 ± 0.02) log *K*_OW_ + (0.01 ± 0.06)	0.78	0.73
(*n* = 689 with 82% log *K*_OC_ ≤ 4.0)	est. log *K*_OW_	85%	98%	87%	log *K*_OC_ = (0.63 ± 0.01) log *K*_OW_ + (0.33 ± 0.05)	0.81	0.68
neutral polar	exp. log *K*_OW_	92%	79%	91%	log *K*_OC_ = (0.74 ± 0.01) log *K*_OW_ + (0.06 ± 0.04)	0.74	0.88
(*n* = 1032with 92% log *K*_OC_ ≤ 4.0)	est. log *K*_OW_	91%	90%	91%	log *K*_OC_ = (0.57 ± 0.01) log *K*_OW_ + (0.30 ± 0.04)	0.67	0.99
anionic or ionizes to anion	exp. log *K*_OW_	98%	57%	95%	log *K*_OC_ = (0.27 ± 0.03) log *K*_OW_ + (1.29 ± 0.07)	0.19	1.36
(*n* = 487 with 93% log *K*_OC_ ≤ 4.0)	est. log *K*_OW_	100%	29%	95%	log *K*_OC_ = (0.20 ± 0.02) log *K*_OW_ + (1.84 ± 0.07)	0.19	1.36
cationic or ionizes to cation	exp. log *K*_OW_	96%	51%	93%	log *K*_OC_ = (0.53 ± 0.03) log *K*_OW_ + (0.64 ± 0.07)	0.37	1.59
(*n* = 607 with 93% log *K*_OC_ ≤ 4.0)	est. log *K*_OW_	100%	7%	93%	log *K*_OC_ = (0.34 ± 0.02) log *K*_OW_ + (2.00 ± 0.09)	0.26	1.74
zwitterionic/amphoteric	exp. log *K*_OW_	100%	14%	91%	log *K*_OC_ = (0.22 ± 0.09) log *K*_OW_ + (1.31 ± 0.25)	0.07	2.04
(*n* = 68 with 91% log *K*_OC_ ≤ 4.0)	est. log *K*_OW_	100%	0%	80%	log *K*_OC_ = (−0.04 ± 0.06) log *K*_OW_ + (1.08 ± 0.30)	0.01	2.11

a Shown are log–log regression statistics and the statistical performance of a log *K*_OW_ or log *D*_OW_ < 4.5 as a screening parameter for the UBA proposed Mobility (M) criteria of log *K*_OC_ < 4.0. rmse = root mean square error.

### Neutral Substances

The log *K*_OC_–log *K*_OW_ correlation for neutral nonpolar substances in Figure 7A is as good as expected based on similar plots reported in the literature.^48,52^ The regression curve for the experimental values was log *K*_OC_ = 0.77 log *K*_OW_ ± 0.01 (*r*^2^ = 0.78, root-mean-square error (rmse) = 0.73), indicating the log *K*_OC_ value was in most cases slightly smaller than the log *K*_OW_ value.^147^ These types of correlations for neutral nonpolar substances have been popular since the 1980s,^148^ though they are generally made for a narrow group of substance classes (e.g., alkanes, PAHs, PCBs, etc.)^147^ and very rarely for many substance classes simultaneously, unless they are necessary to establish linear free energy relationships (LFERs), QSARs, or similar.^48^ The correlation in Figure 7A would not be suitable for LFERs or QSARs, as the individual data points were not checked for their quality but just obtained from the databases using the specified search criteria and data filters. This may explain the high rmse (0.73) and visible outliers. The correlation with estimated log *K*_OW_ values had slightly better statistics for neutral, nonpolar substances, with log *K*_OC_ = 0.63 log *K*_OW_ + 0.33 (*r*^2^ = 0.81, rmse = 0.68). The slight improvements in the correlation statistics may be because estimated *K*_OW_ values already included the same *K*_OC_ in their calibration statistics and because fewer outliers, caused by badly reported experimental data (e.g., unit errors), were present. In both Figure 7A and B, the data gets more scattered for the very large log *K*_OW_ values (those >6.0),^149^ which is anticipated as *K*_OW_ values for such substances are hard to measure accurately and estimation methods would be more prone to extrapolation bias from lack of calibration with such data.

Comparing the log *K*_OC_–log *K*_OW_ relationships for nonpolar and neutral, polar substances (Figure 7A and C), the general range of log *K*_OW_ data shifts from values of 0 to 13 log units to −3 to 9 log units, as expected due to an increased preference for water. As a note, “polarity” is often used synonymously with solubility, but this need not be the case. Very large molecules that are not very soluble (e.g., with a log *K*_OW_ of 9), can be still be considered polar due to the sufficient presence of polar functional groups (consistent with the definition of polarity applied here). The correlation statistics for polar substances are worse than those for the nonpolar substances (log *K*_OC_ = 0.74, log *K*_OW_ + 0.06 (*r*^2^ = 0.74, rmse = 0.88)), as the *r*^2^ value is slightly lower and the rmse is slightly higher. This is partially explained by polar interactions with organic matter and octanol being somewhat different and also variable across diverse soil types.^147^ The correlation statistics obtained when using estimated log *K*_OW_ data for polar substances were slightly worse with log *K*_OC_ = 0.57, log *K*_OW_ ± 0.30 (*r*^2^ = 0.67, rmse 0.99). Looking at Figure 7D, this deviation is due to extremely high estimated log *K*_OW_ values (from 9.0 to 14.0) that correspond with experimental log *K*_OW_ values in Figure 7C that are much lower (< 9.0); this may be related to extrapolation biases from the estimation models.

### Charged and Ionizable Substances

Comparing Figure 7 for neutral substances and Figure 8 for charged and ionizable substances, the difference in log *K*_OC_-–og *K*_OW_/*D*_OW_ correlations is striking, though not unexpected. The ionizable substance correlations in Figure 8 are poor, with *r*^2^ ranging from 0.04 to 0.37 and rmse values ranging from 1.36 to 2.11. However, the data is not randomly distributed despite these poor correlation statistics and some general clustering patterns are evident. When just considering the *K*_OC_–log *K*_OW_ correlation for the ionizable substances (Figure 8a, c, and e), nearly half of the data is clustered between the 1:1 line and 1.5 orders of magnitude below. The relative percentage of substances being ionizable anionic, ionizable cationic, and zwitterionic are 50%, 56%, and 40%, respectively. This area is also where most of the substances clustered for the neutral nonpolar substances (77%) and neutral polar substances (68%,). However, when considering the log *K*_OC_–log *D*_OW_ (minimum between pH 4–9) correlations (Figure 8b, d, and f), the majority of the remaining data is above the 1:1 line for ionizable anionic substances (87%), ionizable cationic substances (93%), and zwitterions (92%), in contrast to neutral nonpolar substances (9%) and neutral polar substances (13%). The obvious mechanistic explanation for why the minimum log *D*_OW_ (pH 4–9) is mostly smaller than log *K*_OC_ is that log *D*_OW_ values only account for an increase solubility in the porewater phase due ionization, but they do not account for the increase in sorption to the soil phase due to ionic interactions. For acids with p*K*_a_ < 4 or conjugated acids with p*K*_a_ > 9, *D*_OW_ can be more than 5 orders of magnitude lower than the neutral form *K*_OW_ within this pH range (based on eqs 3 and 4). Therefore, in general, log *K*_OC_ values are greater than log *D*_OW_ values due to this pH influence. The correlations for anions and cations were not that different, despite soil cationic exchange interactions being generally larger than anion exchange interactions.^150^ For the log *D*_OW_ correlations, this is mainly driven by the pH extrapolations in eqs 3 and 4; and for the minimum log *K*_OW_ correlations of the neutral form, this is likely due to sorption of anion species on average being stronger than the neutral species, similar to cations.

### Screening Thresholds

Figures 7 and 8 show that, for neutral substances, log *K*_OW_ values may be useful for deriving log *K*_OC_ proxy values but not for ionic or ionizable models. However, this does not discount that a log *K*_OW_ or log *D*_OW_ value may be useful as a screening parameter when carrying out a mobility assessment in the absence of log *K*_OC_ data. Previously, fewer data than the current study have been used to concluded that a minimum log *K*_OW_ or minimum log *D*_OW_ < 4.5 could be used as basis for this.^20,21^ Part of the reason for setting the screening log *K*_OW_ threshold at 4.5 is that this is also used for the PBT/vPvB assessment guidelines under REACH,^32^ where it is recommended that if a P/vP substance has a log *K*_OW_ > 4.5, then it should be screened for bioaccumulation potential. Therefore, setting this value as the threshold would prioritize screening for either bioaccumulation or mobility, depending on if the log *K*_OW_ was either above or below 4.5.

In Figures 7 and 8 the “chemical space” for those substances that have a log *K*_OC_ < 4.0 and also have a log *K*_OW_/log *D*_OW_ < 4.5 is plotted. As is evident, many substances do cluster in this chemical space. A comparison of the frequency for which substances with a log *K*_OW_/log *D*_OW_ < 4.5 have a log *K*_OC_ < 4.0 is presented in Table 10 for estimated and experimental values, where it is shown that this occurs for 85% and 88% of the neutral nonpolar substances, respectively; 91% and 92% of the neutral polar substances, respectively; 100% and 98% of the (ionizable) anionic substances, respectively; 100 and 96% of the (ionizable) cationic substances, respectively; and 100% of the zwitterionic/amphoteric substances. The sensitivity in predicting “Not M” correctly using estimated and experimental values was 98% and 91% of the neutral nonpolar substances, respectively; 90% and 79% of the neutral polar substances, respectively. However, the screening criteria are not good at screening for “Not M” ionic substances, as many of the substances with a log *K*_OW_/*D*_OW_ < 4.5 had a log KOC of >3 or >4 due to extra ionic interactions with organic carbon. The sensitivity for predicting “Not M” was 29% and 57% of the (ionizable) anionic substances, respectively; 7% and 51% of the (ionizable) cationic substances, respectively; and, 0% and 15% of zwitterionic substances, respectively. The overall efficiency of this criteria was nevertheless quite high, ranging from 85% to 95% for all ionizable substances, despite the poor sensitivity for predicting “Not M”, as most of the ionic substances had both a log *K*_OC_ < 4.0 and a log *K*_OW_/log *D*_OW_ < 4.5, implying that most ionizable substances would be considered M or vM with these criteria. Based on this good overall efficiency, the screening criteria of log *K*_OW_/log *D*_OW_ is <4.5 is, for the purposes of this study, considered suitable for concluding “Potentially M/vM”, based on the log *K*_OC_ < 4.0 threshold for Mobility, but not “Not M”. As can be assessed visually in Figures 4 and 5, the lower the log *K*_OC_ threshold, the greater the percentage of M/vM substances that will be correctly screened for (e.g., it is rare to see a substance with a log *K*_OC_ < 2.0 and log *K*_OW_/log *D*_OW_ that is >4.5); however, also the lower the log *K*_OC_ threshold, the greater the number of “Not M” substances that will be considered “Potentially M/vM” if the screening value is held constant at log *K*_OW_/log *D*_OW_ < 4.5.

#### Mobility Assessments

**Figure 9:**
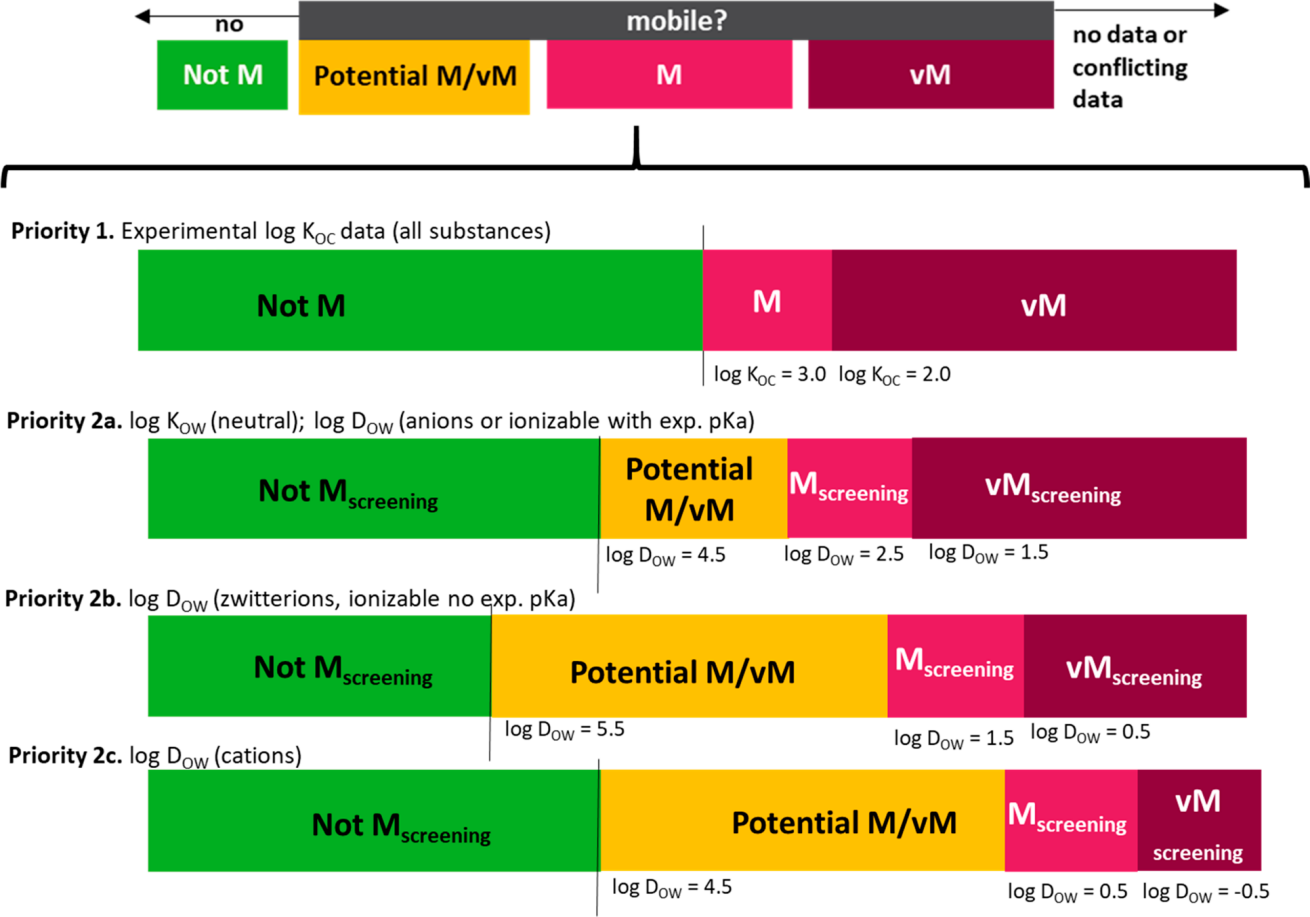
Applied approach toward screening for mobility based on log *D*_OW_ or log *K*_OW_ values for the M/vM assessment in the absence of high-quality log *K*_OC_ data, based on the workflow for the PMT/vPvM assessment (Figure 2). The above suggestion is based on the PMT/vPvM criteria proposed by the EC in 2021. A corresponding figure with the PMT/vPvM criteria proposed by UBA in 2019 is presented in the Supporting Information.

Based on the correlation data and statistics presented Figures 7 and 8 and Table 10, an approach is suggested in Figure 9 for using log *K*_OW_/log *D*_OW_ values to derive the mobility screening categories: “Not M_screening_”, “Potential M/vM”, “M_screening_”, or “vM_screening_”. Priority 1 in Figure 9, which applies to all substance classes, includes minimum log *K*_OC_ values that are experimentally determined using batch tests or similar (pH 4–9). Here the current M/vM criteria proposed by the EC of log *K*_OC_ < 3.0 for M and log *K*_OC_ < 2.0 are used. An alternative version of Figure 9 based on the UBA proposed M/vM criteria can be found in the Supporting Information (Figure S1). Priority 2 is the screening level based on log *K*_OW_/log *D*_OW_ data. Priority 2a applies for neutral substances (between pH 4–9), anions, and ionizable substances with an experimental p*K*_a_ available. Here Potential M/vM is applied to the log *K*_OW_/log *D*_OW_ range between 4.5 and 2.5, M_screening_ for the range between 2.5 and 1.5, and vM_screening_ for <1.5. Priority 2b is applied to zwitterions and ionizable substances with an estimated p*K*_a_, as these are associated with the most uncertain log *K*_OW_/log *D*_OW_ values (e.g., the rmse of 2 orders of magnitude for zwitterions in Table 10 and the uncertainty around predicted p*K*_a_ values in Table 8); these are considered “Potential M/vM” if they have a log *K*_OW_/log *D*_OW_ between 5.5 and 1.5, M_screening_ between 1.5 and 0.5, and vM_screening_ < 0.5. Finally, Priority 2c is used for cations to account for the stronger ionic interacitons with soil; here “Potential M/vM” is a log *K*_OW_/log *D*_OW_ between 4.5 and 1.5, M_screening_ between 0.5 and −0.5, and vM_screening_ < −0.5.

It is noted that, based on the data distribution in Table 10, these screening conclusions are considered conservative, as they are more likely to make “false positive” assessments of M/vM than “false negative” predictions of “Not M”.

As part of the PMT/vPvM hazard assessment workflow (Figure 2), only persistent substances need to be evaluated for mobility. Considering the uncertainties associated with the persistency assessment compared to the mobility assessment, it may make more sense to assess mobility before persistency. However, it is still recommended to assess persistence first, as persistent substances are generally problematic if emitted in high volumes;^40^ therefore, persistent substances should be evaluated for various potential exposure routes, be it as part of a PBT/vPvB assessment, a persistent organic pollutant (POP) assessment, for ozone depletion or other effects.^31,151,152^

**Figure 10:**
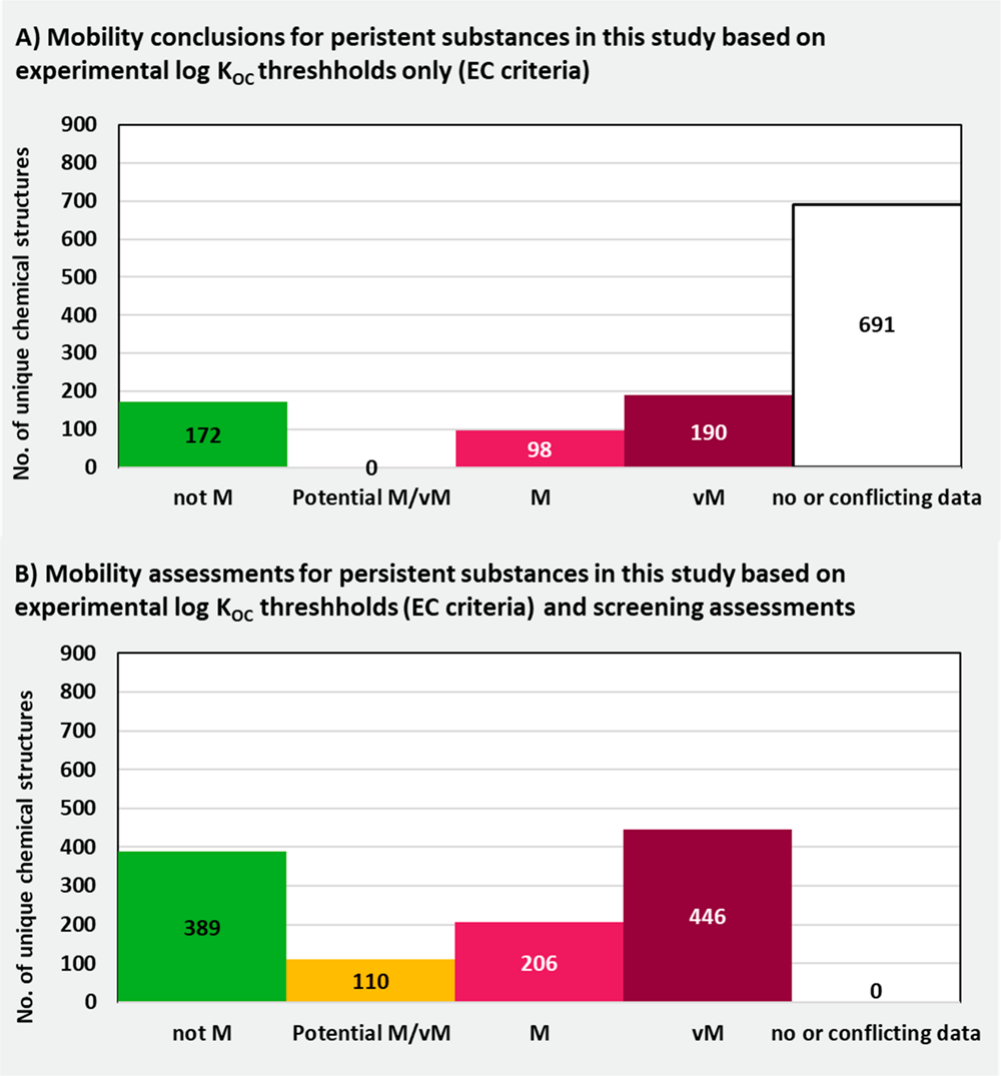
Mobility assessments of the substances considered in this study that were assessed as P, vP, and Potential P/vP++ using (A) the criteria proposed by the EC based on log *K*_OC_ thresholds and (B) the additional screening thresholds based on log *K*_OW_/*D*_OW_ screening parameters in Figure 9.

Figure 10 presents the outcome of mobility assessments for all the 1151 P, vP, and Potential P/vP++ substances assessed in this study, showing the results when only Priority 1 log *K*_OC_ data is used (with the criteria proposed by the EC) and when the Priority 2 screening-based conclusions are considered in addition. As is evident, there were 691 of the 1151 persistent substances for which no log *K*_OC_ was available; however, when allowing the use of the screening parameters (Figure 9), there were no persistent substances for which a mobility assessment could not be made. In either case, the most common mobility assessment conclusions were, in order, vM, "Not M", M, and "Potential M/vM". Consequently, using the log *K*_OW_/log *D*_OW_ screening thresholds presented here would increase the substances considered persistent and mobile based on the proposed criteria from the EC from 288 to 652.

### Toxicity Data

**Figure 11:**
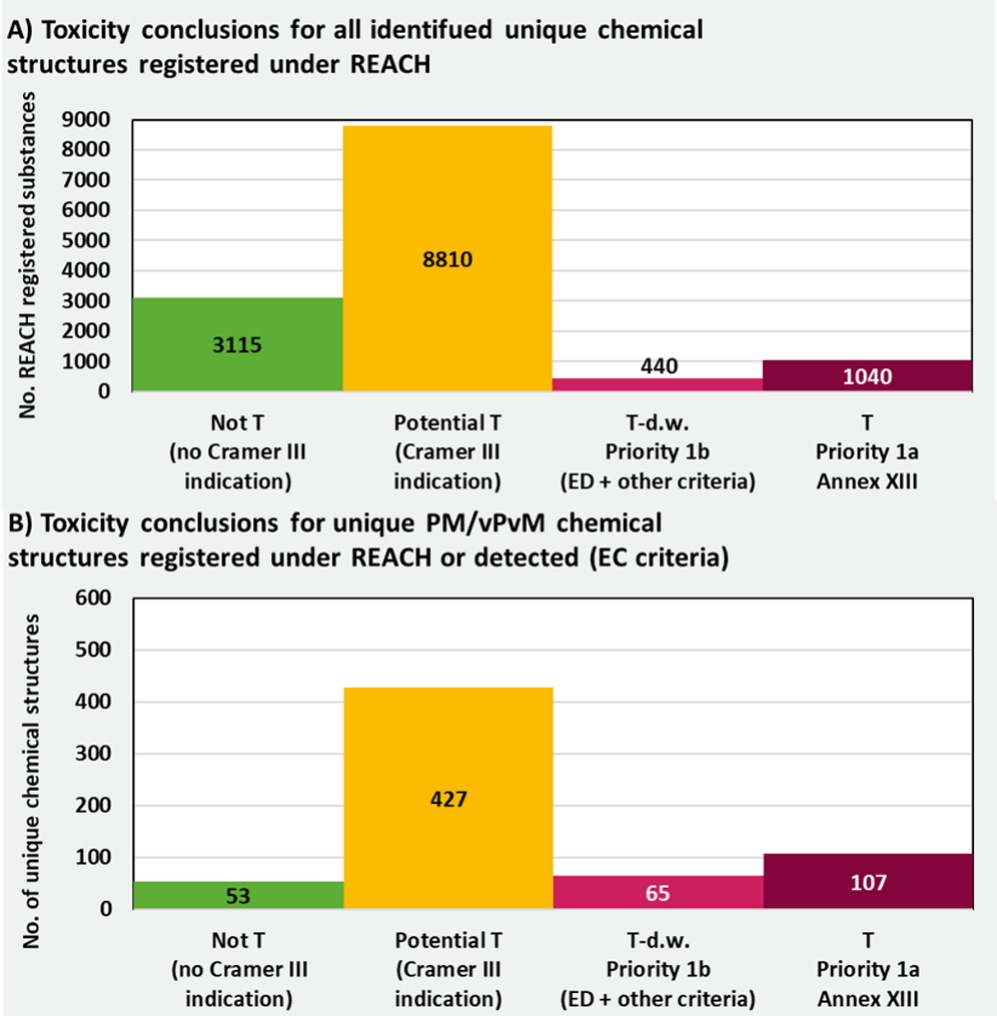
Distribution of toxicity assessments for (A) all identified unique chemical structures registered under REACH or transformation products thereof and (B) all identified persistent and mobile (PM) or very persistent and very mobile (vPvM) chemical structures registered under REACH or detected in the environment.

The compilation of harmonized or broad consensus toxicity assessments for all 13405 REACH registered substances and transformation products thereof is presented in Figure 11A. Based on the REACH Annex XIII criteria that considers toxicity to aquatic organisms and diverse human health end points, 1040 of these substances are considered toxic (Table S1). Considering also the additional toxicity criteria from UBA for PMT substances (described above), there is an additional 440 substances that are considered toxic. Figure 11B presents toxicity assessments for the 652 unique chemical structures registered under REACH and/or detected in the water media considered as PM or vPvM substances based on the proposed criteria from the EC. Of these, 107 are considered toxic according REACH Annex XIII and an additional 65 are considered toxic when using the additional UBA criteria. Of the substances that are not considered toxic, most them have structures that meet the Cramer Class III criterion, implying their structures permit no strong indication of safety and perhaps toxicity.^153^ For the REACH registered substances, 66% of them met the Cramer Class III criterion without a toxic end point identified, and for those considered as PM/vPvM substances, this applied to 67% of them.

### Distribution of PMT/vPvM Hazard Assessments

**Figure 12:**
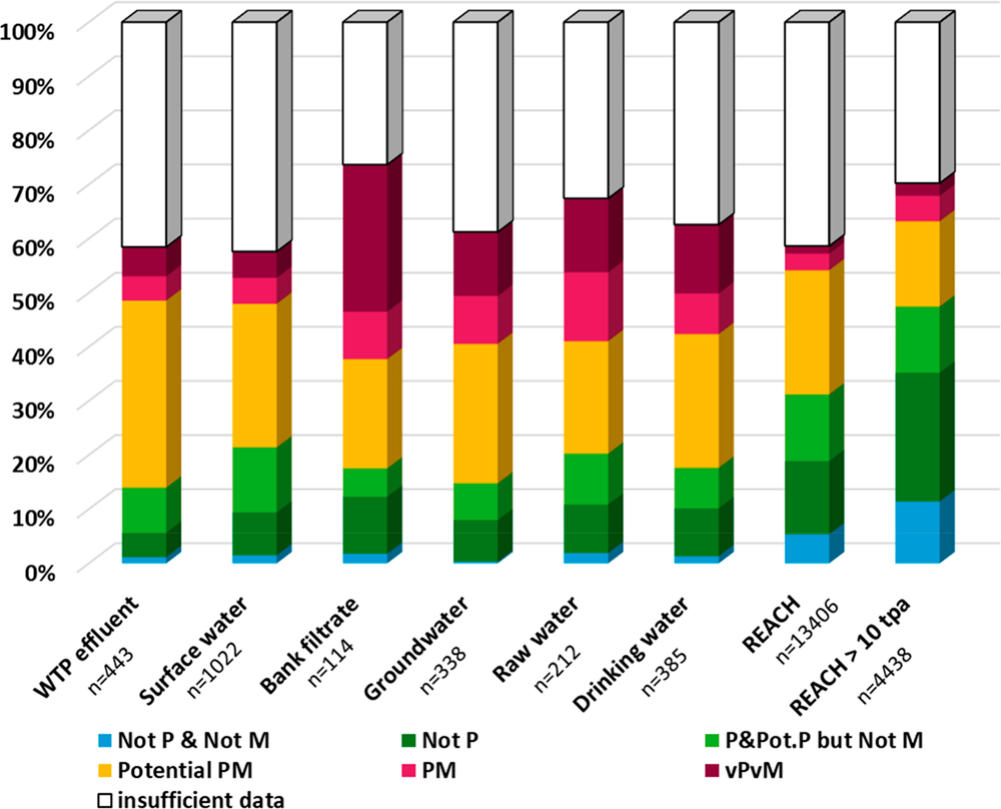
Distribution of all persistence and mobility conclusions among unique chemicals detected in monitoring studies of different freshwater media and REACH registered substances. Assessments are made based on the criteria proposed by the EC. A corresponding figure based on the criteria proposed by the UBA is provided in the Supporting Information.

Figure 12 presents the relative distribution of persistency and mobility assessments based on the criteria proposed by the EC for the substances detected in the different water media as well as those registered under REACH. For the remainder of the text, only the results using the criteria proposed by the EC are presented unless the UBA criteria is explicitly stated.

As evident in Figure 12, there was insufficient data to carry out a persistence and mobility assessment for many of the detected substances (between 26% for bank filtrate to 42% for surface water) and also for the REACH registered substances (between 30% for those registered at >10 tons per annum to 41% for all identified organic constituents). If the Priority 3 weight-of-evidence methods for persistence (Figure 4) or Priority 2 weight-of-evidence methods for mobility (Figure 9) were not included, these numbers would be much larger. Specifically, if persistence assessments were exclusively based on experimental half-life data, readily/inherently biodegradability tests and existing harmonized P evaluations, and mobility assessments were only based on experimental log *K*_OC_ data, PMT/vPvM hazard assessments could only be made for 1067 of the 14203 unique chemicals considered in this study. This means an assessment could not be made for 93% of substances.

A much smaller fraction of REACH registered substances was considered PM or vPvM (total 4%) compared to those detected. Substances in bank filtrate had the highest proportion (36%), followed by raw water (26%), groundwater (21%), drinking water (20%), surface water (10%), and wastewater (10%). This again indicates that the probability of a random substance detected in drinking water or groundwater being a PM or vPvM substance is substantially larger than a random REACH registered substance (by a factor of 5–9). Similarly, REACH registered substances had the most “Not PM” substances (being the total of “Not P & Not M”, “Not P”, “(Pot.) P & Not M”) with 31% of all REACH registered substances and 47% of those registered at >10 tons per annum. The inventory of substances detected in freshwater consisted of 14–20% “Not PM” substances, depending on the media. The “Potential PMT/vPvM” substances comprised a similar percentage of all inventories, being 16% for REACH substances registered at >10 tons per annum (or 23% of all REACH substances) and between 21% and 35% detected in various water media.

Though the highest abundance of PM and vPvM substances in drinking water related media like bank filtrate is expected, the substantial presence of “Not PM” substances in the same media may be unexpected. Their presence can be accounted for by either large emissions, local emissions, lack of favorable conditions for biodegradation, or fast hydraulic flow rates, as mentioned in the Introduction. A closer look at the "Not PM" data in drinking water related media indicates that using the UBA proposed criteria, the majority are "Not P" (Figure S2); though with the proposed EC criteria, there is an increase of "Not M" substances (Figure 12). As examples, there were two “Not M” substances according to the UBA criteria (cholesterol and β-sitosterol) with a log *K*_OC_ > 4.0 reported in bank filtrate,^96^ but these were likely present due to local emissions from naturally occurring sources. There were also 14 “Not P” substances in bank filtrate, all of which were mobile and associated with high emissions. To elaborate, seven of these substances were registered under REACH at tonnages of >1000 tons per annum (therefore potentially high emissions). The others: bisphenol A, triphenyl phosphate, 2-methyl-2*H*-isothiazol-3-one (a popular biocide), methyl cinnamate, toluene-4-sulfonamide (a plasticizer), 2-amino-3,5-xylenesulfonic acid, and 4-dodecyl-benzenesulfonic acid were also likely high production substances.

Similarly for drinking water, there were 15 “Not M” substances that were detected with a log *K*_OC_ > 4.0, which is accounted for by some of them being vP substances (four long-chain PFAS, and two ubiquitous POP substances: hexachlorobenzene and aldrin), substances associated with drinking water contact materials like polyvinyl chloride water pipes (DEHP, 2 alkyl-phenols), and the remainder being pharmaceuticals and personal care products (PPCPs) of unknown production volume (mefenamic acid, fenofibrate, octyl methoxycinnamate, telmisartan). There were also 36 “Not P” substances detected in drinking water, with 16 registered under REACH in 2019 at over 1000 tons per annum (therefore large emissions are likely), with the remainder being previously produced at high production volumes (bisphenol A, butyl benzyl phthalate), being PPCPs of suspected high volume (saccharin, nicotine, ephedrine, estradiol, androstenedione, sodium salicylate, acetylsalicylic acid, theophylline), or being the naturally produced chemical camphor, the plasticizer toluene-4-sulfonamide, and three low sorbing substances of unknown use (dimethylbenzenesulfonic acid, dicholoracetic acid, and 2-chloroethanol). The PMT/vPvM criteria is set up to isolate the compounds the highest propensity to be widely distributed in groundwater and contaminant water extraction points; these "Not PM" substances detected in drinking water are considered less problematic as they would be easier to manage through emission reduction, and likley also water treatment.

**Figure 13:**
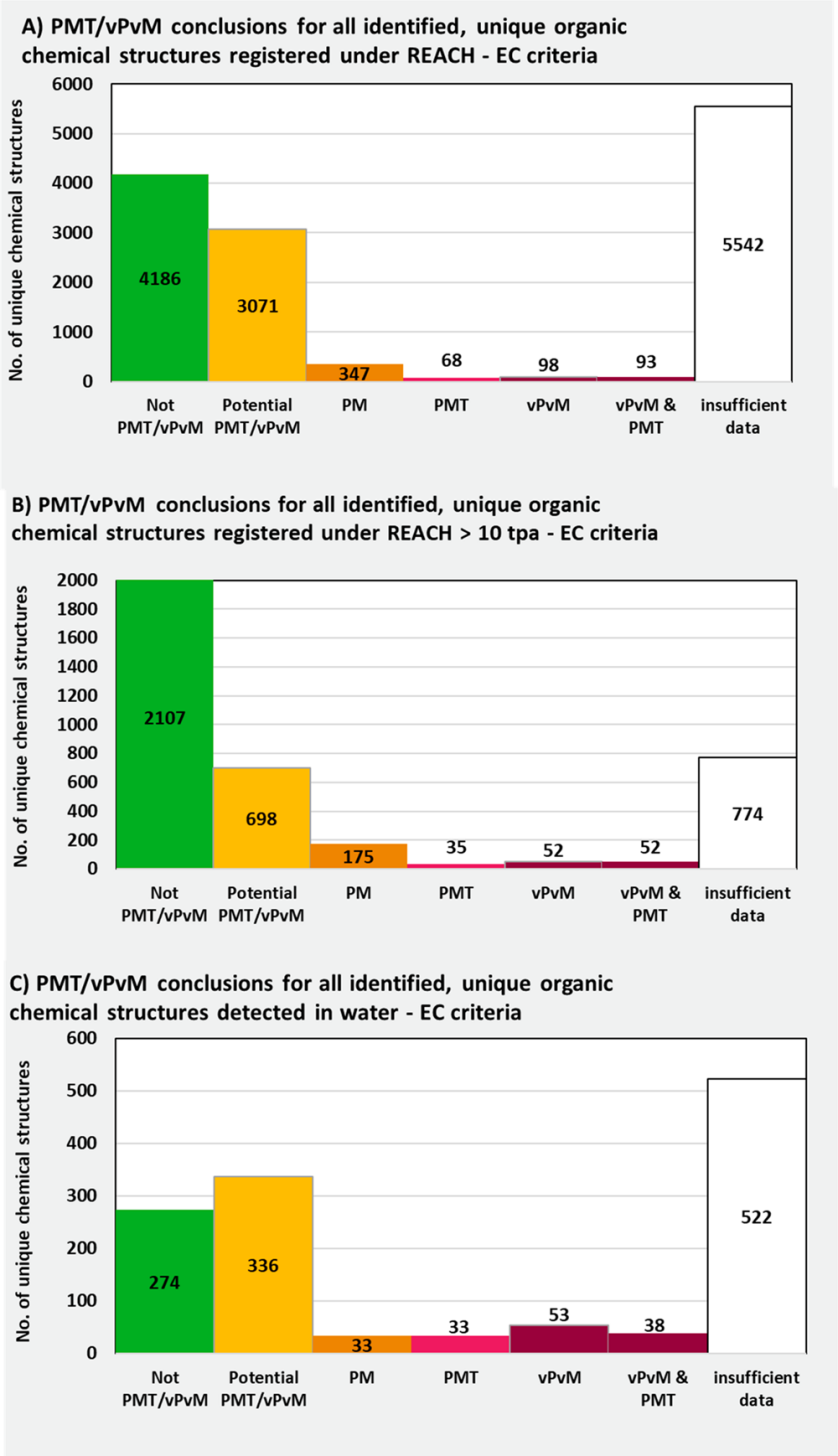
Distribution of PMT/vPvM hazard assessment distributions based on the criteria proposed by the EC and additional screening criteria for (A) all unique chemical structures that were REACH registered in September 2019, (B) those with volumes >10 tons per annum, and (C) unique chemical structures detected in freshwater resources.

Figure 13 presents the overall distribution of PMT/vPvM conclusions based on the criteria proposed by the EC for (A) all unique organic structures among REACH registered substances, (B) those registered at >10 tons per annum, and (C) those detected in water media. The numbers of substances meeting the criteria proposed by the EC and the UBA criteria are presented in Table 11. Comparing Figures 13 and 12, it is evident that many PM substances would not be considered as PMT substances, as they did not fulfill the toxicity criteria. For instance, for the REACH registered substances shown in Figure 13A, only 68 of the 415 PM substances are considered PMT and 93 of the 191 vPvM substances would be considered “vPvM & PMT”. In total, there were 259 REACH registered substances that met the EC’s PMT/vPvM criteria, which corresponds to 1.9% of all identified REACH registered organic constituents. In contrast, for the substances detected in freshwater media, there was a total of 118 substances that meet the EC’s PMT/vPvM criteria, which corresponds to 17.9% of the detected substances. Just considering the substances detected in bank filtrate, groundwater, raw water, and drinking water, 82 substances met these criteria, or 25.5% of the detected substances. This, again, illustrates substances detected in drinking water related media are more persistent and mobile than REACH registered substances, due to environmental degradation and sorption to soils and sediments, as captured by the parameters *t*_1/2_ and *K*_OC_.

**Table 11:** Number of Unique Chemical Constituents Meeting Different PMT/vPvM Conclusions Based on the Criteria Proposed by the EC and the UBA Criteria^a^

	EC/UBA				
	REACH		detected		REACH
PMT/vPvM conclusion	all constituents	>10 tons per annum	all water media	all DW media	all DW media
not PMT/vPvM	4186/3595	2107/1926	274/192	122/85	91/70
potential PMT/vPvM	3071/3504	698/800	336/391	158/171	76/81
PM	347/421	175/201	33/26	21/17	13/12
PMT	68/68	35/37	33/25	27/22	21/18
vPvM	98/131	52/77	53/71	57/71	24/31
vPvM and PMT	93/144	52/78	38/62	34/53	30/43
vPvM or PMT	259/343	139/192	124/158	118/146	75/92
no conclusion/data	5542	774	522	239	56/56
total	13 405	3893	1289	658	311
%PMT/vPvM	2%/3%	4%/5%	10%/12%	17%/22%	24%/30%

a The distribution of conclusions is presented for the unique organic constituents registered under REACH as of September 2019, those registered >10 tons per annum, substances detected in DW media (bank filtrate, groundwater, raw water, drinking water), all water media (additionally surface water and bank filtrate), and REACH substances detected in DW media. Numbers in italic show a decrease in number of substances when the UBA criteria is selected.

#### Thresholds and Sensitivity Analysis

The number of substances that are classified as PMT/vPvM within any given inventory are obviously dependent on (1) the defined thresholds of P, M, and T and (2) the data quality requirements for assessing those thresholds. It is straightforward to conceptualize what the effect of adjusting the P/vP thresholds would be. For instance, increasing threshold half-live values would reduce the number of P/vP substances (and increase the number of “Not P” substances). Not allowing for weight-of-evidence conclusions would expand the number of “Potential P/vP” and “insufficient data” conclusions while decreasing the number of “Not P” and P/vP conclusions. If one were to introduce an alternative persistency threshold instead of media specific half-lives, like the emission scenario dependent multimedia parameter *P*_ov_,^154^ this would either severely restrict the number of substances considered P/vP if intense data requirements are needed, or would have an unknown impact if low data quality modeling threshold values are introduced as weight-of-evidence. If the readily/inherent biodegradability tests were used as the threshold for P, the Potential PMT/vPvM substances would become PM, PMT, or vPvM. This would result in most of the detected substances in freshwater and approximately, a third of the REACH registered substances, being PMT/vPvM substances. Therefore, how the P/vP criteria are parametrized has a substantial impact on the number of PMT/vPvM substances.

Similarly, the sensitivity of adjusting log *K*_OC_ thresholds directly impacts the number of persistent substances meeting the M/vM criteria. A sensitivity analysis of this can be made by looking at the differences in the number of PMT/vPvM substances when using the criteria proposed by the EC and UBA, which differ primarily in their log *K*_OC_ cutoffs, as presented in Table 11. As expected from Table 11, adopting the log *K*_OC_ cutoffs for M/vM of 4.0/3.0 proposed by the UBA, instead of the 3.0/2.0 thresholds as proposed by the EC, the number of “Not PMT/vPvM” substances across all inventories decreases (e.g., by 16% for all identified organic constituents among REACH registered substances and by 43% for the detected substances) and the total number of PMT, vPvM, and “vPvM & PMT” substances increases (e.g., by 24% for REACH registered substances and 22% for detected substances). Further, the number of “Potential PMT/vPvM” substances also increases (e.g., by 12% for REACH registered substances and 14% for detected substances). The only conclusions where sensitivity is not evident or obvious are for the percentages of PM and PMT substances, because some “Not PMT” and “Potential PMT/vPvM” substances will become PM or PMT, while other PM and PMT substances will become “vPvM” and “vPvM & PMT”.

When comparing the criteria proposed by the EC to that proposed by UBA, the UBA proposed criteria results in a higher percentage of substances detected in drinking water related media being classified as PMT/vPvM substances. Overall, 22% of all substances in drinking water related media meet the UBA PMT/vPvM criteria, compared to 17% when using the criteria proposed by the EC, but these percentages could be greater considering that for 37% of substances in drinking water related media there is insufficient data to make a PMT/vPvM conclusion. For substances detected in drinking water related media registered under REACH, there were fewer substances for which there was insufficient data to make a PMT/vPvM assessment (18%), and of these 30% met the UBA criteria compared to 24% that met the EC criteria. Nevertheless, this is a clear indication that substances detected in drinking water relevant media are a factor 10 more likely to meet a PMT/vPvM criteria than the a given substance registered in REACH. For this reason, the PMT/vPvM criteria based on *t*_1/2_ and log *K*_OC_ are considered fit-for-purpose as hazard criteria, as this increase by a factor 10 occurred even without considering substance emissions. Further, as presented above, the majority of “Not PMT/vPvM” substances occurring in drinking water sources appear to be due to widespread or local emissions of “Not P” substances.

Another way to set persistence and mobility thresholds would be to use a function like the GUS index (eq 2 and Figure 1). The impacts of using such a criteria are evident in Figure 1, where the criteria proposed by the EC (as well as the UBA’s vPvM criteria) clearly comprise a smaller range of substances compared to the GUS index “leachers” (GUS > 2.8). However, using this criterion would miss the “nonleachers” that have been detected in sources of drinking water with a log *K*_OC_ between 3.0 and 4.0.

As presented in Table 11, of the 14 203 substances considered in this assessment, there are 298 and 394 PMT/vPvM substances identified when using the criteria proposed by EC and UBA criteria, respectively. If all weight-of-evidence conclusions were removed and only experimental, simulated half-lives and experimental minimum log *K*_OC_ values were considered, there would be 65 and 93 PMT/vPvM substances identified, respectively.

## Environmental Implications

The findings here have implications for the hazard and risk assessment of PMT/vPvM substances and related regulations. The hazard assessment of PMT/vPvM substances refers to whether a substance has the intrinsic substance properties to contaminate water resources over long temporal and spatial scales, even when emitted at low-levels, and serves as a warning to prevent emissions. The risk assessment refers to investigating whether a PMT/vPvM substance could cause deleterious local or regional impacts given its current or planned emissions. Threshold values are currently under discussion in Europe for the PMT/vPvM substance hazard classes, related to the continuum where the longer the *t*_1/2_ and lower the *K*_OC_ sorption, the greater the hazard. As presented above, the combination of *t*_1/2_ and *K*_OC_ are fit-for-purpose to indicate an increased probability of a substance contaminating drinking water resources if emitted; further, they can also be used to indicate increased drinking water purification costs.^7,155^ When certain combinations of *t*_1/2_ and *K*_OC_ thresholds are crossed, chemical regulations (like CLP and REACH) are needed to enable labeling or registration of this hazard to instigate risk management measures, or when necessary authorization or restriction steps, to prevent long-term threats to water resources of such substances. Other regulations, such as agrochemical regulations, industrial emission regulations (e.g., in Europe the Industrial Emissions Directive (2010/75/EU) and the Aarhus Convention), or water quality regulations (e.g., the Urban Waste Water Directive (91/271/EE), Water Framework Directive (2000/60/EC), Groundwater Directive (2006/118/EC) or Drinking Water Directive (2006/118/EC)) can also aim to prevent water resource contamination. Chemical regulations, industrial regulations, and water regulations should ultimately work in synergy to ensure the best risk mitigation strategies for PMT/vPvM substances.

Where regulators set the PMT/vPvM substance thresholds, as well as their data quality requirements, will ultimately impact the number of substances within REACH and the CLP regulation that are considered as PMT/vPvM substances. This has both environmental implications as well as complex socioeconomic implications. The costs associated with the thresholds would mainly be in the form of extra testing that would have to be done for suspected and identified PMT/vPvM substances, developing and implementing risk management measures, and, if this leads to restrictions, potentially redesigning production factories and corresponding supply chains. The benefits to society will come in the form of reduced water remediation costs, health benefits for the general population over intergenerational time scales and thus reduced health care costs, as well as environmental protection from chemical threats.^16^ A further benefit would be the creation of an innovation space for non-persistent, non-toxic substances, or ecofriendly material replacements to chemicals. The costs and benefits are not symmetric across sectors. Currently, without PMT/vPvM hazard classification being enforced, the costs are being felt most directly by the water sector and healthcare sector. If the PMT/vPvM hazard classification is enforced, costs will be transferred primarily to chemical manufacturers. Therefore, harmonized, interlinked dialogue across stakeholders facilitated by regulators is needed to discuss these cost asymmetries.

The work presented here can indirectly provide context to these potential costs and benefits by indicating how many substances could be considered as having PMT/vPvM properties. For instance, the adoption of the proposed EC PMT/vPvM criteria would affect 0.5–2.7% of substances, though the maximum of this range could increase if there was more data for substances considered “Potential PMT/vPvM” or with “insufficient data”. The substantial lack of both *t*_1/2_ data and log *K*_OC_ data for diverse substances is a central challenge for persistence and mobility assessment going forward.

To address the lack of *t*_1/2_ data, there is a need to both simplify experimental methods for determining half-lives as well as to increase the accuracy of QSARs. These two developments are not mutually exclusive, as more experimental data would be invaluable to improve the calibration of persistency QSARs; likewise, the QSARs themselves can be used to form hypotheses toward chemical applicability domains for testing in future experiments. Recently, an approach to simplify *t*_1/2_ testing in water, compared to the OECD 309 guideline, was proposed.^156^ This approach demonstrated that substituting (expensive) ^14^C-labeled compounds with nonradiolabeled aniline was suitable for benchmarking half-lives.^156^ This method was applied to a group of seven previously suspected PMT/vPvM substances that were all later confirmed to be persistent in water.^155,156^ PMT/vPvM hazard assessments based on weight-of-evidence here could be further prioritized for persistency testing using this simplified method.

For mobility assessments, too, more experimental data and better models are needed, though primarily for charged and ionizable organic compounds. For neutral substances, experimental *K*_OC_ measurements, like OECD 106, are straightforward and are available for a large variety of functional groups. Further, PP-LFERs for estimating *K*_OC_ work quite well to fill the data gap for neutral chemicals within their chemical applicability domains; therefore, a remaining, though straightforward, data gap to fill would be analyzing *K*_OC_ data for neutral substance classes that are outside the current chemical applicability domains in order to expand them. For charged and ionizable substances, the situation is more complex.^61^ In order to address this, advanced mechanistic and systematic sorption studies on studies on diverse soils, accounting for variations in ion exchange capacity, soil composition (organic carbon and mineral content), and porewater chemistry (competing ions, pH), are needed to improve *K*_OC_ predictive models. In the meantime, while such research is underway, the minimum log *K*_OC_ value from batch tests remains the most robust and conservative way to approach a hazard assessment (though local *K*_OC_ or *K*_D_ values should be used for local risk assessment).^61^

Despite the data gaps in *t*_1/2_ and *K*_OC_ data, when data are available, they are considered fit-for-purpose for defining PMT/vPvM thresholds. These criteria can identify the substances that are the most problematic to freshwater resources over large time and spatial scales even if emitted at low-levels. Further, good-quality screening parameters, such as the better performing persistency QSARs, and high-quality *K*_OW_/*D*_OW_ data can be used to close data gaps through weight-of-evidence approaches (Figures 4 and 9) for making initial PMT/vPvM assessments until better quality experimental *t*_1/2_ and *K*_OC_ data are available.

Using currently proposed thresholds, between 1.9% and 2.6% of REACH registered substances were identified as PMT/vPvM, compared to between 24 and 30% of substances detected in drinking water sources. The list of identified PMT/vPvM substances in this study, as presented in Table S1, could be further explored in follow-up studies, including assessing the impacts of PMT/vPvM regulations, prioritizing substances for water monitoring, cataloging the uses of these substances, prioritizing persistency and mobility test measurements, refining risk assessment procedures and developing risk governance strategies. Due to the potentially large number of PMT/vPvM substances already in commerce and likely undetected in drinking water sources, concerted efforts are needed by researchers, regulators, and industry to better understand and manage the threats these hazardous substances pose. This will ultimately protect the sources of our drinking water for future generations

## Supporting Information Available

All data used in this report; suggested and applied approach towards screening for mobility based on log *D*_OW_ or log *K*_OW_ values for the M/vM assessment in the absence of high-quality log *K*_OC_ data using the proposed UBA criteria from 2019; distribution of all PMT/vPvM conclusions amongst unique chemicals detected in monitoring studies and amongst REACH registered substances using the propose UBA criteria from 2019 (XLSX)
